# Effect of small heat release and viscosity on thermal-diffusive instability

**DOI:** 10.1038/s41598-021-99163-6

**Published:** 2021-10-12

**Authors:** Keigo Wada

**Affiliations:** Articulation Center for High School and University, Kanazawa University, Kakuma, Kanazawa, Ishikawa 920-1192 Japan

**Keywords:** Engineering, Mathematics and computing, Physics

## Abstract

The linear stability of a thermal reaction front has been investigated based on the thermal-diffusive model proposed by Zel’dovich and Frank-Kamenetskii, which is called ZFK model. In the framework of ZFK model, heat-conduction and mass-diffusion equations are treated without the effect of hydrodynamic flow. Then, two types of instability appear: cellular and oscillatory instabilities. The cellular instability has only positive real part of growth rate, while the oscillatory instability is accompanied with non-zero imaginary part. In this study, the effect of heat release and viscosity on both instabilities is investigated asymptotically and numerically. This is achieved by coupling mass-conservation and Navier–Stokes equations with the ZFK model for small heat release. Then, the stable range of Lewis number, where the real part of growth rate is negative, is widened by non-zero values of heat release for any wavenumber. The increase of Prandtl number also brings the stabilization effect on the oscillatory instability. However, as for the cellular instability, the viscosity leads to the destabilization effect for small wavenumbers, opposed to its stabilization effect for moderate values of wavenumber. Under the limit of small wavenumber, the property of viscosity becomes clear in view of cut-off wavenumber, which makes the real part of growth rate zero.

## Introduction

The analysis of thermal propagation of a flame front has been performed so far based on the thermal-diffusive model, or the ZFK model, proposed by Zel’dovich and Frank-Kamenetskii^1,2^. In the framework of ZFK model, the effect of hydrodynamic flow is successfully decoupled from the heat and mass-diffusion equations. This is achieved by the assumption of small difference between the values of density, or temperature, evaluated at burned and unburned edges of reaction front^3^. In this case, we only need to treat the heat-conduction and mass-diffusion equations accompanied with the exothermic reaction term, which is usually expressed by the Arrhenius type^4–6^. The thickness of reaction front is characterized by the inverse of Zel’dovich number^7^. By assuming the large values of Zel’dovich number, the analysis of inner structure of reaction front, where the reaction term is confined, becomes possible^8,9^. The values of Zel’dovich number larger than 2 has been found to be valid in the context of burning-rate eigenvalue problem^10–13^. The inner structure of reaction front has been investigated based on the method of matched asymptotic expansions^14^. Then, the boundary conditions across the reaction front are obtained for temperature and mass fraction. We do not pursue the analysis of previous research, and instead, utilize the boundary conditions already derived^15^. We note that the hydrodynamic flow is also considered in our study. Therefore, the boundary conditions of hydrodynamic quantities across the reaction front are also required and they are presented in the previous work^16^.

The linear stability analysis of the thermal-diffusive model without hydrodynamic flow has been performed for one reactant^17^, for two reactants^18^, for the case of heat loss^19^ and in the context of ignition temperature^20^. The nonlinear treatment of this model is also discussed based on Hopf bifurcation^21^. The thermal-diffusive instability is categorized into two types, the cellular and oscillatory instabilities, depending on values of Lewis number^22^. The stability boundary of thermal-diffusive instability is usually determined with respect to Lewis number and wavenumber. The cellular instability is observed for some range of Lewis number less than unity. The stability boundary of cellular instability shows monotonic decrease of Lewis number as the wavenumber increases. On the other hand, that of oscillatory instability is characterized by the non-monotonic behavior of Lewis number as a function of wavenumber. These features are obtained as a result of neglecting the effect of thermal expansion, or of no heat release. Our result shows that, for the stability boundary of cellular instability, the existence of heat release changes the monotonic behavior of Lewis number to the non-monotonic one.

This study focuses on the pure thermal-diffusive instability. Therefore, the boundary conditions across the reaction front are different from those used in the hydrodynamic model. Strictly speaking, in the hydrodynamic model, a flame front is treated as a zero-thickness interface and the basic flow, which is assumed to be steady one-dimensional flow, is described by the constant density and velocity, which are discontinuous across the front. Such a discontinuity is the intrinsic property of hydrodynamic instability, or the Darrieus–Landau instability (DLI): the DLI claims that the growth rate of perturbations is an linear function of wavenumber, whose coefficient is positive due to the heat release, and a planar flame front is unstable for any wavenumber. As a consequence of the jump of normal velocity across a flame front, the tangential components of velocity field induced by small perturbations are also discontinuous. This fact is essential for the existence of DLI. However, our study is performed in the scale of thermal thickness of a flame front, or in the framework of thermal-diffusive model. Then, the boundary conditions across the reaction front, which have been derived based on the large activation energy asymptotics, are described by the continuity of all quantities except for the pressure, whose discontinuity is supported by the viscosity. Due to the continuity of normal velocity, the tangential components of velocity becomes continuous for small perturbations. For a basic flow, the reaction front is considered as a density continuous interface, whereas the derivative of density is discontinuous. As a result, the DLI does not appear in our treatment.

In this study, we consider the case of small heat release. Then, we need to deal with not only the thermal-diffusive model but also hydrodynamic one. So far, the coupling of these two models has been achieved by the multi-scale analysis based on the thermal thickness of a flame front, or by introducing the preheat zone inside the front^23–26^. This approach successfully combines the DLI^27–29^ with the Markstein effect which reflects the thermal and mass diffusivities^30–32^. The numerical study to understand the dynamics of a flame front has been performed for cellular and non-cellular fronts^33,34^ and for cellular front with heat loss^35–37^. Despite of many works on the instability of a flame front, the influence of heat release and viscosity on the stability boundaries of cellular and oscillatory instabilities has not ever been declared clearly. Besides, the effect of higher order terms of wavenumber, such as third and forth order terms, on the growth rate has not been discussed in detail. The existence of third order term of wavenumber has the possibility of leading to two cut-off wavenumbers, opposed to the case of zero heat release, where the cut-off wavenumber is determined uniquely: the cut-off wavenumber means the value of wavenumber at which the growth rate of perturbations changes its sign. In order to reveal these influence, we directly examine the linear stability of reaction front for infinitesimal perturbations with the heat release being a small parameter. Besides, the calculation of numerical solutions is performed based on the TVD-MacCormack scheme^38–44^.

## Methods

We employ the following non-dimensional governing equations^13,25^

1 $$\begin{aligned} \frac{\partial \rho }{\partial t} + \nabla \cdot (\rho \varvec{V})&= 0, \end{aligned}$$

2 $$\begin{aligned} \rho \left( \frac{\partial \varvec{V}}{\partial t} + (\varvec{V}\cdot \nabla )\varvec{V}\right)&= -\frac{1}{\gamma {Ma}^2}\nabla P_M + Pr\left( \Delta \varvec{V} + \frac{1}{3}\nabla (\nabla \cdot \varvec{V})\right) , \end{aligned}$$

3 $$\begin{aligned} \rho \left( \frac{\partial {T}}{\partial t} + (\varvec{V}\cdot \nabla ){T}\right)&= \Delta T + qQ, \end{aligned}$$

4 $$\begin{aligned} \rho \left( \frac{\partial {Y}}{\partial t} + (\varvec{V}\cdot \nabla ){Y}\right)&= \frac{1}{Le}\Delta Y - Q, \end{aligned}$$

5 $$\begin{aligned} P&= \rho T , \end{aligned}$$

where the non-dimensional variables, $\rho$, $\varvec{V}=(U,V,W)$, *T*, $P_M$ and *Y* are the density of mixture, velocity field, temperature, pressure and mass fraction of a deficient species which is consumed through the exothermic chemical reaction. The parameters $\gamma$, *Ma*, *Pr*, *q* and *Le* are the specific heat ratio, Mach number, Prandtl number, heat release and Lewis number, respectively. The dependent variables are made dimensionless by use of those of fresh mixture at a position far from a flame front, where the flow field is assumed to be steady planar, denoted by $\tilde{\rho }_{-\infty }$, $\tilde{S}_{L}$, $\tilde{T}_{-\infty }$, $\tilde{P}_{-\infty }$ and $\tilde{Y}_{-\infty }$. Especially, $\tilde{S}_{L}$ is called the laminar flame speed, or the burning velocity, which is the propagation velocity of a planar flame front into a quiescent fuel mixture^13^. Hereafter, the variables with tilde symbol stand for dimensional ones.

6 $$\begin{aligned} \rho&= \frac{\tilde{\rho }}{\tilde{\rho }_{-\infty }},\quad&T&= \frac{\tilde{T}}{\tilde{T}_{-\infty }},\quad&P_M&= \frac{\tilde{P}}{\tilde{P}_{-\infty }},&Y&= \frac{\tilde{Y}}{\tilde{Y}_{-\infty }},\quad&\varvec{V}&= \frac{\tilde{\varvec{V}}}{\tilde{S}_{L}}, \nonumber \\ \tilde{l}_d&= \frac{\tilde{D}_{th}}{\tilde{S}_{L}},&Le&= \frac{\tilde{D}_{th}}{\tilde{D}},&Pr&=\frac{\tilde{\mu }\tilde{c}_p}{\tilde{\lambda }},\quad&\tilde{D}_{th}&= \frac{\tilde{\lambda }}{\tilde{\rho }_{-\infty }\tilde{c}_p}, \quad&Ma&= \frac{\tilde{S}_{L}}{\tilde{c}_s}. \ \ \end{aligned}$$

The parameters $\tilde{l}_d$, $\tilde{D}_{th}$, $\tilde{D}$, $\tilde{\mu }$, $\tilde{c}_p$ and $\tilde{\lambda }$ are the thermal thickness of a flame front, thermal diffusivity, diffusion coefficient of a deficient species, coefficient of viscosity, specific heat at constant pressure and thermal conductivity. We assume that $\tilde{D}$, $\tilde{\mu }$, $\tilde{c}_p$ and $\tilde{\lambda }$ are all constant. The adiabatic sound speed is defined by $\tilde{c}_s=(\gamma \tilde{P}_{-\infty }/\tilde{\rho }_{-\infty })^{1/2}$. We normalize the coordinates $\tilde{\varvec{x}}$ and the time $\tilde{t}$, by use of $\tilde{l}_d$ and $\tilde{S}_L$, as

7 $$\begin{aligned} \varvec{x}=\tilde{\varvec{x}}/\tilde{l}_d,\quad t=\tilde{t}\tilde{S}_L/\tilde{l}_d. \end{aligned}$$

We introduce the Cartesian coordinate system $\varvec{x}=(x,y,z)$, with an unperturbed steady planar reaction front lying on the *x*–*y* plane and propagating in the negative direction of *z*-axis.

According to the previous work^25^, the reaction term *Q* is expressed by the following form.

8 $$\begin{aligned} Q = \frac{\Lambda \rho Y}{\varepsilon ^2}\exp \left( \frac{T_b}{\varepsilon }\frac{T-T_b}{T}\right) . \end{aligned}$$

The parameter $\varepsilon$ is defined by

9 $$\begin{aligned} \varepsilon =\frac{T_b^2}{N}= \frac{q}{\beta }, \end{aligned}$$

where $T_b=1+q$ and $N = \tilde{E} / \tilde{R}_g \tilde{T}_{-\infty }$ are the adiabatic temperature, which is evaluated on the burned side of a reaction front, and the non-dimensional activation energy with the dimensional activation energy $\tilde{E}$ and universal gas constant $\tilde{R}_g$. The Zel’dovich number $\beta =\tilde{E}(\tilde{T}_{b}-\tilde{T}_{-\infty }) / (\tilde{R}_g \tilde{T}_{b}^2)$ will be employed to compare our result with the one obtained in previous works^3,32,45^. Under the limit of large activation energy ($N\rightarrow +\infty$), $\varepsilon$ becomes a small parameter which characterizes the thickness of reaction front. Although the burning-rate eigenvalue $\Lambda$ is important to determine the laminar flame speed^4,5,10–12^, its treatment is out of scope of this work. In order to investigate the effect of heat release and viscosity on the thermal-diffusive instability, we only need to utilize the jump conditions across the reaction front derived in previous works by considering the inner structure of the front^15,16^.

### Coordinate system attached to a flame front

We recall the coordinate system attached to the flame front, $z=F(x,y,t)$, by introducing the following variables^25^.

10 $$\begin{aligned} x = x,\quad y = y,\quad \xi = z - F(x,y,t),\quad t=t. \end{aligned}$$

By use of (10), governing Eqs. (1)–(4) are rewritten as

11 $$\begin{aligned} \frac{\partial \rho }{\partial t} + \nabla _t\cdot (\rho \varvec{V}_t) + \frac{\partial M}{\partial \xi }&= 0, \end{aligned}$$

12 $$\begin{aligned} \rho \frac{\partial \varvec{V}_t}{\partial t} + \rho (\varvec{V}_t\cdot \nabla _t)\varvec{V}_t + M\frac{\partial \varvec{V}_t}{\partial \xi }&= -\frac{\nabla _tP_M}{\gamma {Ma}^2} + \frac{\nabla _tF}{\gamma {Ma}^2}\frac{\partial P_M}{\partial \xi } \nonumber \\&\quad + Pr\left\{ \Delta _{\xi } \varvec{V}_t + \frac{1}{3}\left( \nabla _t-\nabla _tF\frac{\partial }{\partial \xi } \right) \left( \nabla _t\cdot \varvec{V}_t +\frac{\partial S}{\partial \xi }\right) \right\} , \end{aligned}$$

13 $$\begin{aligned} \rho \frac{\partial W}{\partial t} + \rho (\varvec{V}_t\cdot \nabla _t)W + M\frac{\partial W}{\partial \xi }&= -\frac{1}{\gamma {Ma}^2}\frac{\partial P_M}{\partial \xi } + Pr\left\{ \Delta _{\xi } W + \frac{1}{3} \frac{\partial }{\partial \xi } \left( \nabla _t\cdot \varvec{V}_t +\frac{\partial S}{\partial \xi }\right) \right\} , \end{aligned}$$

14 $$\begin{aligned} \rho \frac{\partial T}{\partial t} + \rho (\varvec{V}_t\cdot \nabla _t)T + M\frac{\partial T}{\partial \xi }&= \Delta _{\xi } T + qQ, \end{aligned}$$

15 $$\begin{aligned} \rho \frac{\partial Y}{\partial t} + \rho (\varvec{V}_t\cdot \nabla _t)Y + M\frac{\partial Y}{\partial \xi }&= \frac{1}{Le}\Delta _{\xi } Y - Q, \end{aligned}$$

where *S* denotes the longitudinal velocity of gases relative to a flame front.

16 $$\begin{aligned} S = W - \frac{\partial F}{\partial t} - (\varvec{V}_t\cdot \nabla _t) F. \end{aligned}$$

The tangential components of velocity field are expressed by $\varvec{V}_t=(U,V,0)$. Based on (16), the mass flux is defined by

17 $$\begin{aligned} M=\rho S. \end{aligned}$$

The transverse derivative is denoted by $\nabla _t=(\partial /\partial x, \partial /\partial y, 0)$, and we also introduce the notation $\nabla _t^2=\partial ^2/\partial x^2+ \partial ^2/\partial y^2$. Then, the Laplacian is written as

18 $$\begin{aligned} \Delta _{\xi } = (1+|\nabla _t F|^2)\frac{\partial ^2 }{\partial \xi ^2}+\nabla _t^2-(\nabla _t^2 F)\frac{\partial }{\partial \xi }-2(\nabla _t F)\cdot \nabla _t\frac{\partial }{\partial \xi }. \end{aligned}$$

The unit normal on the reaction layer, which is directed toward the burned side ($\xi >0$), is defined by

19 $$\begin{aligned} \varvec{n} = \frac{\nabla \xi }{|\nabla \xi |} = \frac{1}{\sqrt{1+|\nabla _tF|^2}}\left( -\frac{\partial F}{\partial x},-\frac{\partial F}{\partial y},1\right) . \end{aligned}$$

### Boundary conditions

In the far field from a flame front on the unburned side, where the flow is assumed to be steady planar, all quantities become unity due to the definition (6).

20 $$\begin{aligned} \rho = T = P_M = Y = W = 1,\quad U = V = 0\quad {\text {as}}\quad \xi \rightarrow -\infty . \end{aligned}$$

The deficient component of combustible mixture is assumed to be depleted during the exothermic chemical reaction inside the reaction front. Then, the mass fraction *Y* is equivalently zero on the burned side.

21 $$\begin{aligned} Y \equiv 0 \quad (\xi >0). \end{aligned}$$

In this study, we employ the zero-Mach-number approximation, $Ma\ll 1$. Under this limit, as readily found from (2), the pressure is assumed to have the following asymptotic form^22^.

22 $$\begin{aligned} P_M = 1 + \gamma {Ma}^2P. \end{aligned}$$

In the subsequent discussion, we consider the variation of pressure at $O({Ma}^2)$.

In order to know the profile of physical quantities outside the reaction front, we need the boundary conditions on it. In the limit of large activation energy, $\varepsilon (=q/\beta )\ll 1$, the following jump conditions across the reaction front have been derived^15,16^.

23 $$\begin{aligned}{}[(\varvec{n}\cdot \nabla )M]&=0, \end{aligned}$$

24 $$\begin{aligned}{} [M]=[\varvec{V}\cdot \varvec{n}]=\left[ P-\frac{4}{3} Pr(\varvec{n}\cdot \nabla )(\varvec{V}\cdot \varvec{n})\right]&=0, \end{aligned}$$

25 $$\begin{aligned}{} [{\varvec {n}} \times ({\varvec {V}} \times {\varvec {n}})] =\varvec{0}, \end{aligned}$$

26 $$\begin{aligned}{} [T] =[Y]=\left[ (\varvec{n}\cdot \nabla )\left( T+\frac{q}{Le}Y\right) \right] =0, \end{aligned}$$

27 $$\begin{aligned} (\varvec{n}\cdot \nabla )T|_{\xi =0-}&=q\exp \left( \frac{T|_{\xi =0+}-T_b}{2\varepsilon }\right) , \end{aligned}$$

where the brackets for arbitrary function $\Phi (x,y,\xi ,t)$ means the difference of its values on the burned and unburned sides, $[\Phi ]=\Phi |_{\xi =0+}-\Phi |_{\xi =0-}$.

In the subsequent calculations, the boundary conditions (20), (21), (23)–(27) are imposed to determine the integral constants of unperturbed and perturbed solutions of (1)–(5) for a weakly corrugated front, whose amplitude is infinitesimally small.

### Basic flow

As a basic flow without perturbations, we consider a steady planar flow. The notation of over-bar is used to denote this flow field. For example, the velocity of basic flow is denoted by $\bar{W}=\bar{W}(\xi )$. In this case, the jump conditions (24)–(26) are given by

28 $$\begin{aligned}{}[\bar{M}]=[\bar{W}]=\left[ \bar{P}-\frac{4}{3}Pr\frac{d \bar{W}}{d \xi }\right] =[\bar{T}]=[\bar{Y}]=\left[ \frac{d}{d \xi }\left( \bar{T}+\frac{q}{Le}\bar{Y}\right) \right] =0. \end{aligned}$$

We note that (23) and (27) are automatically satisfied by the solutions obtained by use of (28). For a steady planar flow, the mass conservation Eq. (11) becomes

29 $$\begin{aligned} \frac{d \bar{M}}{d \xi } = 0. \end{aligned}$$

From (29), by use of (20) and (28), we readily find that the steady planar mass flux is unity on both sides of reaction front.

30 $$\begin{aligned} \bar{M} = 1. \end{aligned}$$

Then, the other governing Eqs. (5), (13)–(15) are written down, outside the reaction layer ($\xi <0$, $\xi >0$), as

31 $$\begin{aligned} \frac{d \bar{W}}{d \xi }&= - \frac{d \bar{P}}{d \xi } + \frac{4}{3} Pr\frac{d^2 \bar{W}}{d \xi ^2}, \end{aligned}$$

32 $$\begin{aligned} \frac{d \bar{T}}{d \xi }&= \frac{d^2 \bar{T}}{d \xi ^2}, \end{aligned}$$

33 $$\begin{aligned} \frac{d \bar{Y}}{d \xi }&= \frac{1}{Le}\frac{d^2 \bar{Y}}{d \xi ^2}, \end{aligned}$$

34 $$\begin{aligned} 1&= \bar{\rho }\bar{T}. \end{aligned}$$

We note that the reaction term, included in (14) and (15), is confined inside the reaction front due to the assumption of large activation energy, $\varepsilon \ll 1$, with $T<T_b$ in the zero-Mach-number limit^25^. Subject to (20), (21) and (28), the solutions of unperturbed governing equations (31)–(34) are obtained as

35 $$\begin{aligned} &\bar{T}= \left\{ \begin{array}{lll} 1+q{\text {e}}^{\xi } &{} (\xi<0)\\ 1+q &{} (\xi> 0) \end{array} \right. ,\,\, \bar{Y}= \left\{ \begin{array}{lll} 1-{\text {e}}^{Le\xi } &{} (\xi<0)\\ 0 &{} (\xi> 0) \end{array} \right. ,\,\, \\ & \bar{P}= \left\{ \begin{array}{lll} \left( \frac{4}{3}Pr-1\right) q{\text {e}}^{\xi } &{} (\xi <0)\\ -q &{} (\xi > 0) \end{array} \right. , \,\, \bar{\rho } = \frac{1}{\bar{T}} ,\,\, \bar{W} = \bar{T} . \end{aligned}$$

These solutions are used to calculate small perturbations for a weakly corrugated reaction front below.

### Formulation of perturbations

We consider the situation where the reaction front is slightly deviated from its unperturbed state. We assume that the heat release is sufficiently small.

36 $$\begin{aligned} q\ll 1. \end{aligned}$$

Based on (36), the amplitude of weakly corrugated front is given in the form of harmonic perturbation by

37 $$\begin{aligned} F = q^{2}{\text {e}}^{{\text {i}}k_xx+{\text {i}}k_yy+\omega t}, \end{aligned}$$

where $\omega$ is the growth rate and the wavenumber *k* is defined by $k=(k_x^2+k_y^2)^{1/2}$. The non-dimensionalization is made as $\omega =\tilde{\omega }\tilde{l}_d/\tilde{S}_L$ and $k=\tilde{k}\tilde{l}_d$ with $\tilde{\omega }$ and $\tilde{k}$ dimensional quantities.

We impose small perturbations on the basic flow as follows.

38 $$\begin{aligned} \rho = \bar{\rho } + \rho ',\quad W = \bar{W} + W',\quad \varvec{V}_t = \varvec{V}_t',\quad P = \bar{P} + P',\quad T = \bar{T} + T',\quad Y = \bar{Y} + Y', \end{aligned}$$

where the order of each perturbation is assumed to be

39 $$\begin{aligned} \rho ' = \rho _p(\xi )qF,\ W' = W_p(\xi )qF,\ \varvec{V}_t' = (U_p(\xi ),V_p(\xi ),0)qF,\ P' = P_p(\xi )qF,\ T' = T_p(\xi )qF \sim O(q^{3}), \end{aligned}$$

40 $$\begin{aligned} Y'&= Y_p(\xi )F&\sim O(q^{2})&. \end{aligned}$$

Upon (37)–(40), the perturbed governing equations have the following form with $O(q^4)$ terms omitted due to (36).

41 $$\begin{aligned} \left( \frac{\partial }{\partial t} + \frac{\partial }{\partial \xi }\right) \rho ' - \frac{\partial F}{\partial t}\frac{d \bar{\rho }}{d \xi } + \nabla _t\cdot \varvec{V}_{t}' + \frac{\partial W'}{\partial \xi }&= 0, \end{aligned}$$

42 $$\begin{aligned} \left( \frac{\partial }{\partial t} + \frac{\partial }{\partial \xi }\right) \varvec{V}_t'&= -\nabla _t P' + (\nabla _tF)\frac{d \bar{P}}{d \xi }+ Pr\left( \nabla _t^2+\frac{\partial ^2 }{\partial \xi ^2}\right) \varvec{V}_t' \nonumber \\&\quad + Pr\left\{ \frac{1}{3}\nabla _t\left( \nabla _t\cdot \varvec{V}_t'+\frac{\partial W'}{\partial \xi }\right) -\frac{1}{3}(\nabla _tF)\frac{d^2 \bar{W}}{d \xi ^2}\right\} , \end{aligned}$$

43 $$\begin{aligned} \left( \frac{\partial }{\partial t} + \frac{\partial }{\partial \xi }\right) W' - \frac{\partial F}{\partial t}\frac{d \bar{W}}{d \xi }&= -\frac{\partial P'}{\partial \xi } + Pr\left( \nabla _t^2+\frac{\partial ^2 }{\partial \xi ^2}\right) W' \nonumber \\&\quad + Pr\left\{ - (\nabla _t^2F)\frac{d \bar{W}}{d \xi } + \frac{1}{3}\frac{\partial }{\partial \xi }\left( \nabla _t\cdot \varvec{V}_t'+\frac{\partial W'}{\partial \xi }\right) \right\} , \end{aligned}$$

44 $$\begin{aligned} \left( \frac{\partial }{\partial t} + \frac{\partial }{\partial \xi }\right) T' - \frac{\partial F}{\partial t}\frac{d \bar{T}}{d \xi }&= \left( \nabla _t^2+\frac{\partial ^2 }{\partial \xi ^2}\right) T' - (\nabla _t^2F)\frac{d \bar{T}}{d \xi }, \end{aligned}$$

45 $$\begin{aligned} \left( \frac{\partial }{\partial t} + \frac{\partial }{\partial \xi }\right) Y' -(1-q{\text {e}}^{\xi })\frac{\partial F}{\partial t}\frac{d \bar{Y}}{d \xi } + \left( \rho ' + W'\right) \frac{d \bar{Y}}{d \xi }&= \frac{1}{Le}\left( \nabla _t^2+\frac{\partial ^2 }{\partial \xi ^2}\right) Y' - \frac{1}{Le}(\nabla _t^2F)\frac{d \bar{Y}}{d \xi }, \end{aligned}$$

46 $$\begin{aligned} 0&= \rho '+T'. \end{aligned}$$

As is readily confirmed from (35), the derivatives of $\bar{\rho }$, $\bar{P}$, $\bar{W}$ and $\bar{T}$ are *O*(*q*) and that of $\bar{Y}$ is *O*(1). Unlike the ordinary thermal-diffusive model, the influence of flow field appears in (45) due to the existence of small heat release. In this case, the coupling of hydrodynamic equations and thermal-diffusive ones is possible because the heat equation remains unchanged from the ordinary one and solvable. The detail of calculation is given in the succeeding subsection.

### Calculation of perturbations

We calculate perturbations (39) and (40) by solving perturbed governing Eqs. (41)–(46). From (46), we find that once the perturbation of temperature is calculated, that of density is obtained.

47 $$\begin{aligned} \rho _p = -T_p. \end{aligned}$$

Substituting (35), (37) and (39) into the perturbed heat-conduction Eq. (44), the following differential equation is obtained.

48 $$\begin{aligned} \frac{d^2 T_{p}}{d \xi ^2} - \frac{d T_{p}}{d \xi } - (\omega + k^2) T_{p} =\left\{ \begin{array}{lll} -\left( \omega +k^2\right) {\text {e}}^{\xi } &{} (\xi < 0)\\ 0 &{} (\xi >0) \end{array} \right. . \end{aligned}$$

Assuming $Re[\omega ]>0$, the requirement that perturbations are bounded in space leads to the solution of (48) as

49 $$\begin{aligned} T_{p} = \left\{ \begin{array}{lll} \theta _{m}{\text {e}}^{\lambda _{+}\xi } + {\text {e}}^{\xi } &{} (\xi < 0)\\ \theta _{p}{\text {e}}^{\lambda _{-}\xi } &{} (\xi >0) \end{array} \right. , \end{aligned}$$

where

50 $$\begin{aligned} \lambda _{\pm } = \frac{1\pm \sqrt{1+4(\omega + k^2)}}{2}. \end{aligned}$$

We note that the signs are in the same order. The integral constants $\theta _{m}$ and $\theta _{p}$ are determined in the succeeding subsection.

Next, in order to calculate $P_p$, we transform the perturbed mass conservation Eq. (41) into

51 $$\begin{aligned} \nabla _t\cdot \varvec{V}_{t}' + \frac{\partial W'}{\partial \xi } = \left( \frac{\partial }{\partial t}+\frac{\partial }{\partial \xi }\right) T'-q{\text {e}}^{\xi }\frac{\partial F}{\partial t}, \end{aligned}$$

where (35) and (46) are used and higher order terms with respect to *q* are omitted due to (36). Then, taking the transverse derivative $\nabla _t\cdot$ of (42) and differentiating (43) with respect to $\xi$, and combining them with the help of (51), we obtain

52 $$\begin{aligned} \left( \nabla _t^2+\frac{\partial ^2 }{\partial \xi ^2}\right) P'&= \left( \frac{4}{3} Pr\left( \nabla _t^2+\frac{\partial ^2 }{\partial \xi ^2}\right) -\frac{\partial }{\partial t}-\frac{\partial }{\partial \xi }\right) \left( \left( \frac{\partial }{\partial t}+\frac{\partial }{\partial \xi }\right) T'-q{\text {e}}^{\xi }\frac{\partial F}{\partial t}\right) \nonumber \\&\quad +\left( \frac{\partial F}{\partial t}-\frac{4}{3} Pr(\nabla _t^2F)\right) \frac{d^2 \bar{W}}{d \xi ^2}+(\nabla _t^2F)\frac{d \bar{P}}{d \xi }. \end{aligned}$$

By use of (35), (37) and (39), the combined equation (52) becomes the following differential equation for $P_p$.

53 $$\begin{aligned} \frac{d^2 P_p}{d \xi ^2}-k^2P_p=\left\{ \begin{array}{lll} \left( \frac{4}{3}Pr-1\right) \left( (\lambda _++\omega )^2\theta _m{\text {e}}^{\lambda _+\xi }+\left( 1-k^2\right) {\text {e}}^{\xi }\right) &{} (\xi < 0)\\ \left( \frac{4}{3}Pr-1\right) (\lambda _-+\omega )^2\theta _p{\text {e}}^{\lambda _-\xi } &{} (\xi >0) \end{array} \right. . \end{aligned}$$

Solving (53), we obtain

54 $$\begin{aligned} P_p=\left\{ \begin{array}{lll} p_m{\text {e}}^{k\xi }+\left( \frac{4}{3}Pr-1\right) (\lambda _++\omega )\theta _m{\text {e}}^{\lambda _+\xi }+\left( \frac{4}{3}Pr-1\right) {\text {e}}^{\xi } &{} (\xi < 0)\\ p_p{\text {e}}^{-k\xi }+\left( \frac{4}{3}Pr-1\right) (\lambda _-+\omega )\theta _p{\text {e}}^{\lambda _-\xi } &{} (\xi >0) \end{array} \right. , \end{aligned}$$

where $p_m$ and $p_p$ are integral constants.

The perturbations of velocity field are calculated by substituting (35), (37), (39), (49), (51) and (54) into the perturbed Navier–Stokes equations (42) and (43) as follows.

55 $$\begin{aligned} U_p&=\left\{ \begin{array}{lll} u_m{\text {e}}^{l_+\xi }-\frac{{\text {i}}k_x}{\omega +k}p_m{\text {e}}^{k\xi }+{\text {i}}k_x\theta _m{\text {e}}^{\lambda _+\xi }\qquad \,\,\,\, &{} (\xi < 0)\\ u_p{\text {e}}^{l_-\xi }-\frac{{\text {i}}k_x}{\omega -k}p_p{\text {e}}^{-k\xi }+{\text {i}}k_x\theta _p{\text {e}}^{\lambda _-\xi } &{} (\xi >0) \end{array} \right. , \end{aligned}$$

56 $$\begin{aligned} V_p&=\left\{ \begin{array}{lll} v_m{\text {e}}^{l_+\xi }-\frac{{\text {i}}k_y}{\omega +k}p_m{\text {e}}^{k\xi }+{\text {i}}k_y\theta _m{\text {e}}^{\lambda _+\xi }\qquad \,\,\,\,\, &{} (\xi < 0)\\ v_p{\text {e}}^{l_-\xi }-\frac{{\text {i}}k_y}{\omega -k}p_p{\text {e}}^{-k\xi }+{\text {i}}k_y\theta _p{\text {e}}^{\lambda _-\xi } &{} (\xi >0) \end{array} \right. , \end{aligned}$$

57 $$\begin{aligned} W_p&=\left\{ \begin{array}{lll} w_m{\text {e}}^{l_+\xi }-\frac{k}{\omega +k}p_m{\text {e}}^{k\xi }+\lambda _+\theta _m{\text {e}}^{\lambda _+\xi }+{\text {e}}^{\xi } &{} (\xi < 0)\\ w_p{\text {e}}^{l_-\xi }+\frac{k}{\omega -k}p_p{\text {e}}^{-k\xi }+\lambda _-\theta _p{\text {e}}^{\lambda _-\xi } &{} (\xi >0) \end{array} \right. , \end{aligned}$$

where

58 $$\begin{aligned} l_{\pm } = \frac{1\pm \sqrt{1+4 Pr(\omega + Pr k^2)}}{2 Pr}, \end{aligned}$$

and $u_m$, $u_p$, $v_m$, $v_p$, $w_m$ and $w_p$ are integral constants.

The perturbation of mass fraction is calculated by integrating (45) by use of (35), (37), (40), (47), (49) and (57).

59 $$\begin{aligned} Y_p=\left\{ \begin{array}{lll} c_m{\text {e}}^{\Gamma _+\xi }-Le{\text {e}}^{Le\xi }-\frac{qLe^2\omega }{1-k^2+Le(1-\omega )}{\text {e}}^{(1+Le)\xi }-\frac{q Le^2(\lambda _+-1)}{(1+Le)\lambda _++(1-Le)\omega }\theta _m{\text {e}}^{(\lambda _++Le)\xi }&{}\\ -\frac{qLe^2Pr}{(1+LePr)l_++(1-LePr)\omega }w_m{\text {e}}^{(l_++Le)\xi } + \frac{qLe k}{k^2-\omega ^2}p_m{\text {e}}^{(k+Le)\xi } &{} (\xi < 0)\\ 0 &{} (\xi >0) \end{array} \right. , \end{aligned}$$

where

60 $$\begin{aligned} \Gamma _{\pm } = Le\frac{1\pm \sqrt{1+4(\omega + k^2/Le)/Le}}{2}, \end{aligned}$$

and $c_m$ is an integral constant. Unlike the classical thermal-diffusive model, the solution of perturbed mass fraction (59) includes the terms reflecting the effect of hydrodynamic flow due to non-zero values of heat release *q*. Thanks to these terms, it becomes possible to incorporate the influence of heat release and viscosity into the thermal-diffusive instability.

At last, by substituting solutions (49), (55)–(57) into the mass conservation equation (51), we obtain the following relations.

61 $$\begin{aligned} {\text {i}}k_xu_m+{\text {i}}k_yv_m+l_+w_m = {\text {i}}k_xu_p+{\text {i}}k_yv_p+l_-w_p = 0. \end{aligned}$$

### Dispersion relation

In this subsection, we calculate the dispersion relation of thermal-diffusive instability, which represents the relation between the growth rate and wavenumber for perturbations, by substituting the solutions obtained above into the boundary conditions across the reaction front. For the perturbed quantities, the boundary conditions (23)–(27) are written down as

62 $$\begin{aligned} \frac{d \bar{\rho }}{d \xi }\Big |_{-}(W'|_{-}-\omega F)+\bar{\rho }|_{-}\frac{\partial W'}{\partial \xi }\Big |_{-}+\frac{\partial \rho '}{\partial \xi }\Big |_{-}\bar{W}|_{-}+\rho '|_{-}\frac{d \bar{W}}{d \xi }\Big |_{-}&=\bar{\rho }|_{+}\frac{\partial W'}{\partial \xi }\Big |_{+}+\frac{\partial \rho '}{\partial \xi }\Big |_{+}\bar{W}|_{+}, \end{aligned}$$

63 $$\begin{aligned} U'|_{-} = U'|_{+},\quad V'|_{-}&= V'|_{+}, \end{aligned}$$

64 $$\begin{aligned} W'|_{-}&= W'|_{+}, \end{aligned}$$

65 $$\begin{aligned} P'|_{-}-\frac{4}{3}Pr\frac{\partial W'}{\partial \xi }\Big |_{-}&=P'|_{+}-\frac{4}{3}Pr\frac{\partial W'}{\partial \xi }\Big |_{+}, \end{aligned}$$

66 $$\begin{aligned} T'|_{-} = T'|_{+},\quad \frac{\partial T'}{\partial \xi }\Big |_{-}&= \frac{\beta }{2}T'|_{-}, \end{aligned}$$

67 $$\begin{aligned} Y'|_{-}&= 0, \end{aligned}$$

68 $$\begin{aligned} \frac{\partial T'}{\partial \xi }\Big |_{-} + \frac{1}{Le}\frac{\partial Y'}{\partial \xi }\Big |_{-}&= \frac{\partial T'}{\partial \xi }\Big |_{+}. \end{aligned}$$

We note that the continuity condition of mass flux in (24) leads to the continuity of density perturbation due to (64), but this gives no information. In fact, this is the same condition as the first one in (66) because of perturbed equation of state (46). From (61)–(67), we determine integral constants of perturbations. After that, (68) is used to calculate the dispersion relation of thermal-diffusive instability including the effect of small heat release accompanied with the viscosity.

At first, the integral constants for thermal-diffusive quantities are easily determined. Indeed, substituting (49) into (66), we obtain

69 $$\begin{aligned} \theta _{m} + 1 = \theta _{p},\quad \lambda _{+}\theta _m + 1 = \frac{\beta }{2}\theta _p. \end{aligned}$$

Then, integral constants $\theta _{m}$ and $\theta _{p}$ of temperature perturbation are determined by solving (69) as

70 $$\begin{aligned} \theta _{m} = \frac{\beta -2}{2\lambda _+-\beta },\quad \theta _p = 2\frac{\lambda _+-1}{2\lambda _+-\beta }. \end{aligned}$$

The expression of $c_m$ is obtained from (67), by use of (59), as

71 $$\begin{aligned} c_m=Le+\frac{q\omega Le^2}{1-k^2+Le(1-\omega )}+\frac{qLe^2(\lambda _+-1)}{(1+Le)\lambda _++(1-Le)\omega }\theta _m +\frac{qLe^2Pr}{(1+LePr)l_++(1-LePr)\omega }w_m-\frac{qLek}{k^2-\omega ^2}p_m. \end{aligned}$$

If $q=0$, the above solutions (70) and (71) coincides with those in the classical thermal-diffusive model^22,32^.

Next, the integral constants for hydrodynamic flow field are determined. The substitution of (55) and (56) into (63) gives

72 $$\begin{aligned} & u_m-\frac{{\text {i}}k_x}{\omega +k}p_m+{\text {i}}k_x\theta _m = u_p-\frac{{\text {i}}k_x}{\omega -k}p_p+{\text {i}}k_x\theta _p,\\ &  v_m-\frac{{\text {i}}k_y}{\omega +k}p_m+{\text {i}}k_y\theta _m = v_p-\frac{{\text {i}}k_y}{\omega -k}p_p+{\text {i}}k_y\theta _p. \end{aligned}$$

From (61) and (72), $u_m$, $u_p$, $v_m$ and $v_p$ are eliminated to give the following relation.

73 $$\begin{aligned} -l_+w_m+\frac{k^2}{\omega +k}p_m- k^2\theta _m = -l_-w_p+\frac{k^2}{\omega -k}p_p- k^2\theta _p. \end{aligned}$$

The continuity of normal velocity (64), with (57) substituted, leads to

74 $$\begin{aligned} w_m-\frac{k}{\omega +k}p_m+\lambda _+\theta _m + 1 = w_p+\frac{k}{\omega -k}p_p+\lambda _-\theta _p. \end{aligned}$$

Solving (73) and (74), we find

75 $$\begin{aligned} p_m&= \frac{\omega +k}{2k^2}\left( (k+l_+)w_m-(k+l_-)w_p+(\lambda _++k) k\theta _m-(\lambda _-+k) k\theta _p+k\right) , \end{aligned}$$

76 $$\begin{aligned} p_p&= \frac{\omega -k}{2k^2}\left( (k-l_+)w_m-(k-l_-)w_p+(\lambda _+-k) k\theta _m-(\lambda _--k) k\theta _p+k\right) . \end{aligned}$$

By use of solutions (54) and (57), the boundary condition of pressure (65) is transformed into

77 $$\begin{aligned}&p_m+\left( \frac{4}{3}Pr-1\right) \left( (\lambda _++\omega )\theta _m+1\right) -\frac{4}{3}Pr\left( l_+w_m-\frac{k^2}{\omega +k}p_m+\lambda _+^2\theta _m + 1\right) \nonumber \\&\quad =p_p+\left( \frac{4}{3}Pr-1\right) (\lambda _-+\omega )\theta _p -\frac{4}{3}Pr\left( l_-w_p-\frac{k^2}{\omega -k}p_p+\lambda _-^2\theta _p\right) . \end{aligned}$$

From (75)–(77), we obtain

78 $$\begin{aligned} w_p = \frac{l_+\omega +k^2}{l_-\omega +k^2}w_m. \end{aligned}$$

The continuity condition of derivative of mass flux (62) is reduced, by use of (50), (69) and (73) while being careful about $1/(1+q)\approx 1-q+O(q^2)$, to

79 $$\begin{aligned} w_m-\frac{k}{\omega +k}p_m+\left( 3\lambda _+\omega +1\right) \theta _m+2\omega +4=(2\lambda _-+\omega )\theta _p. \end{aligned}$$

Substituting (70) and (75) into (79), with the help of (78), we get

80 $$\begin{aligned}  w_m & = \frac{l_-\omega +k^2}{(\omega -k)(l_--l_+)-2(l_-\omega +k^2)}\frac{2}{2\lambda _+-\beta }\\ & \quad \times \left( -\frac{\beta }{2}(5\lambda _-+k+2\omega )+\lambda _-+(k+2\omega )\lambda _++3(\omega + k^2)\right) . \end{aligned}$$

Finally, by use of (49), (59), (70), (71) and (80), the dispersion relation is calculated from (68) as follows.

81 $$\begin{aligned}&\left( (\lambda _+-\lambda _-)\left( 1+\frac{q}{2}\frac{k+\Gamma _-}{k-\omega }\right) +\frac{qLe\lambda _-(\lambda _++\Gamma _-)}{(1+Le)\lambda _++(1-Le)\omega }\right) \frac{\beta -2}{2\lambda _+-\beta }\\&+\lambda _+-\Gamma _-+\frac{q}{2}\frac{k+\Gamma _-}{k-\omega }\left( \lambda _+-k\right) \nonumber  -(1+\Gamma _-)\frac{qLe\omega }{1-k^2+Le(1-\omega )}\\&-q\left( \frac{LePr(l_++\Gamma _-)}{(1+LePr)l_++(1-LePr)\omega }+\frac{k+\Gamma _-}{2}\frac{l_--l_+}{l_-\omega +k^2}\right) w_m=0. \end{aligned}$$

The numerical and analytical studies of (81) are given below.

## Asymptotic results

### The case of $q=0$

In the pure thermal-diffusive model, or the ZFK model^1,2^, the effect of hydrodynamic flow was effectively decoupled from the heat and mass-diffusion equations. This situation is easily recovered by taking $q=0$ in (81). It may be helpful to recall the original dispersion relation for thermal-diffusive instability^3,32^, which is given by

82 $$\begin{aligned} (\lambda _+-\lambda _-)\frac{\beta -2}{2\lambda _+-\beta }+\lambda _+-\Gamma _-=0. \end{aligned}$$

In addition, resorting to the assumption that the Lewis number is nearly equal to unity, which is acceptable for real gases^6,13^, we introduce *O*(1) constant *le* as

83 $$\begin{aligned} Le=1+\frac{le}{\beta }, \end{aligned}$$

under the limit of large Zel’dovich number, $\beta \gg 1$. By use of (83), the dispersion relation (82) is transformed into the following form.

84 $$\begin{aligned} \left( 1-\sqrt{1+4(\omega +k^2)}\right) (1+4(\omega +k^2))=-\frac{le}{2}\left( 1+2\omega -\sqrt{1+4(\omega +k^2)}\right) . \end{aligned}$$

The numerical calculation of (84) has been shown and summarized by many works^3,22,32^. The cellular instability is observed for some range of Lewis number less than unity and the oscillatory one appears for large values of Lewis number. The cellular instability is characterized by the positive values of real part of growth rate without imaginary part and the oscillatory one is by those with imaginary part. This tendency remains unchanged for the case of non-zero values of heat release as discussed below.

### Computation of dispersion relation

We consider the case of $q\ne 0$. In order to compare our result with the previous research, we apply the assumption (83) to the dispersion relation (81) under $\beta \gg 1$. Then, we obtain the reduced dispersion relation, with $q\beta = O(1)$ and *O*(*q*) terms omitted, as follows.

85 $$\begin{aligned}&\frac{\omega +\lambda _-}{1-2\lambda _+}le+2\lambda _-(1-2\lambda _+)-\frac{q\beta }{2}\left( \frac{k+\lambda _-}{k-\omega }+\frac{\lambda _-}{\omega +k^2}+2\frac{\omega (1+\lambda _-)}{\omega +k^2-2}\right) \nonumber \\&\quad +q\beta \frac{k+2\omega +5\lambda _-}{2kPr(l_+-k)-k-\omega }\left( \frac{k+\lambda _-}{2}(1-2Prl_+)+Pr^2\frac{(l_++\lambda _-)(k^2+l_-\omega )}{l_+(1+Pr)-(Pr-1)\omega }\right) =0. \end{aligned}$$

**Figure 1:**
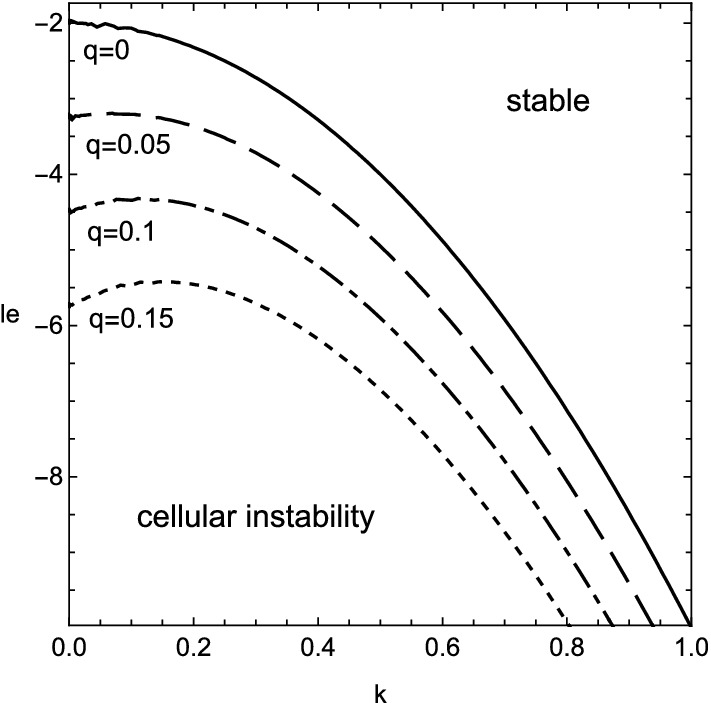
Stability boundary of cellular instability for several values of heat release, $q=0$ (solid line), 0.05 (dashed), 0.1 (dot-dashed) and 0.15 (dotted), with $\beta =10$ and $Pr=0$.

**Figure 2:**
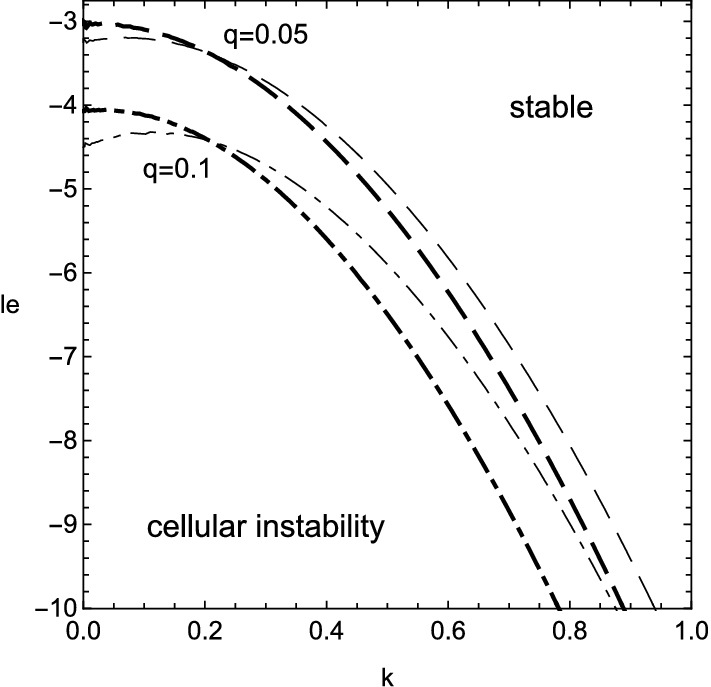
The same as Fig. 1 but for two values of Prandtl number, $Pr=0$ (thin lines) and 3/4 (thick lines).

**Figure 3:**
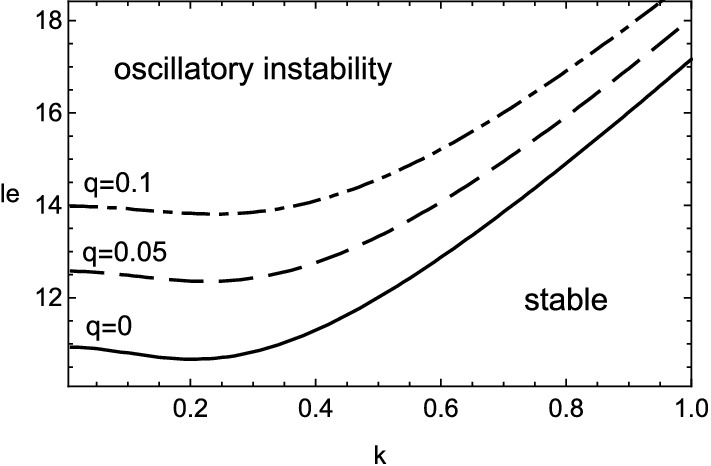
Stability boundary of oscillatory instability for several values of heat release, $q=0$ (solid line), 0.05 (dashed) and 0.1 (dot-dashed), with $\beta =10$ and $Pr=0$.

**Figure 4:**
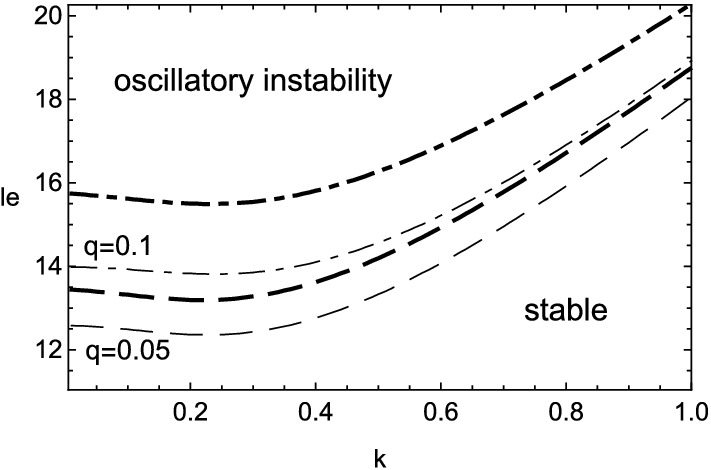
The same as Fig. 3 but for two values of Prandtl number, $Pr=0$ (thin lines) and 3/4 (thick lines).

The numerical results of (85) are shown in Figs. 1, 2, 3 and 4 for $\beta =10$. The stable range of Lewis number, where the real part of growth rate takes negative values, is greatly altered from that in the previous case of $q=0$ due to the heat release accompanied with the viscous effect. At first, from Figs. 1 and 3, the inclusion of heat release is found to extend the stable domain for both cellular and oscillatory instabilities. In addition, Fig. 4 shows that the non-zero values of Prandtl number, or the viscous effect, also broadens the stable range of Lewis number for the oscillatory instability. Contrary to such stabilization effect, it should be emphasized that the viscosity has the destabilizing effect on the cellular instability if the wavenumber is small, as shown in Fig. 2. For some moderate values of wavenumber, the cellular instability is stabilized by the viscosity.

### Cellular instability for small wavenumber and growth rate

As discussed above, we find that the viscosity brings the destabilization effect on the cellular instability for small values of wavenumber, opposed to the stabilizing one for finite values. Therefore, it may be meaningful to investigate the dispersion relation (81) in detail for small wavenumbers to reveal the destabilization effect of viscosity. For this purpose, we consider two cases: $\omega \sim k^2\ll 1$ and $\omega \sim k^4\ll 1$. Especially, in the latter case, our result contains the new effect of third order term with respect to wavenumber in the reduced dispersion relation.

#### The case of $\omega \sim k^2\ll 1$

At first, the effect of wavenumber is examined up to second order term, which corresponds to the Markstein effect reflecting the curvature effect^30,31,45^. By assuming that $\omega \sim k^2\ll 1$, the dispersion relation (81) is reduced to

86 $$\begin{aligned} 2(1+q\beta )\omega +\left( \frac{2+(Le-1)\beta }{Le}+2q\beta +\frac{q\beta (1-Pr)}{(1+Le)(1+LePr)}\right) k^2=0. \end{aligned}$$

For $q=0$, the above Eq. (86) is consistent with that obtained in the previous work^45^. In addition, assuming (83) for $\beta \gg 1$ with $q\beta =O(1)$, the reduced dispersion relation (86) gives the following growth rate.

87 $$\begin{aligned} \omega =\frac{-k^2}{2(1+q\beta )}\left( 2+le+\frac{q\beta }{2}\frac{5+3Pr}{1+Pr}\right) . \end{aligned}$$

The growth rate (87) clearly shows how the variation of *le* in the range of small wavenumber influences the cellular instability with small heat release. The stability condition of (87), or $\omega <0$, is given by $le>-2-q\beta (5+3Pr)/(2+2Pr)$. The stability boundary $le=-2$, which is obtained in the previous research^3,32,45^, is extended by the inclusion of heat release *q* accompanied with the viscous effect represented by Prandtl number *Pr*. The increase of *q* leads to the relaxation of lower bound of *le*. On the other hand, the rise of *Pr* narrows the stable range of *le*. This result explains the destabilizing effect of viscosity for small wavenumber as implied by the numerical computation in Fig. 2.

#### The case of $\omega \sim k^4\ll 1$

Next, we proceed to higher order terms of wavenumber, $O(k^3)$ and $O(k^4)$ terms. We note that $O(k^3)$ term is intrinsic to the effect of heat release and not included in the original dispersion relation of thermal-diffusive model^3,32^. In the present case, the cut-off wavenumber is no longer determined uniquely and there is the possibility of two cut-off wavenumbers depending on values of Lewis number.

Based on the assumption of $\omega \sim k^4\ll 1$, the dispersion relation (81) is transformed into

88 $$\begin{aligned}&2(1+q\beta )\omega +\left( \frac{2+(Le-1)\beta }{Le}+2q\beta +\frac{q\beta (1-Pr)}{(1+Le)(1+LePr)}\right) k^2+\frac{q\beta }{Le}\left( 2-Pr-\frac{5-2Pr}{1+LePr}\right) k^3\nonumber \\&\quad +\left( \frac{2}{Le}-\frac{(Le^3-1)(\beta -2)}{Le^3}-2q\beta -q\beta \frac{Pr}{Le}\frac{(5-2Pr)(1-LePr)-LePr^2}{1+LePr}\right) k^4=0. \end{aligned}$$

Solving the reduced dispersion relation (88), by use of the assumption (83) for $\beta \gg 1$ with $q\beta =O(1)$, we find

89 $$\begin{aligned} \omega =\frac{1}{2(1+q\beta )}(\omega _2k^2+\omega _3k^3+\omega _4k^4), \end{aligned}$$

with

$$\begin{aligned} &  \omega _2 =-\left( 2+le+\frac{q\beta }{2}\frac{5+3Pr}{1+Pr}\right) ,\quad \omega _3 =q\beta \left( Pr+\frac{3-4Pr}{1+Pr}\right) \ge 0,\\ & \omega _4 =3le-2+q\beta \frac{2+7Pr(1-Pr)+Pr^3}{1+Pr}. \end{aligned}$$

We note that the Prandtl number *Pr* typically takes positive and small values: for example, $Pr\approx 3/4$ in air^13^. For $q=0$, the $O(k^4)$ term in (89) is consistent with previous work^45^, which treated the case of $le\approx -2$, that is $\omega _4k^4/2\approx -4k^4$. For non-zero values of heat release, as confirmed from $\omega _2$ and $\omega _3$, the rise of Prandtl number increases the growth rate at second order of wavenumber and decreases it at third order. Because the wavenumber is assumed to be small, the viscous effect is not so important at the forth order of wavenumber. By setting $\omega =0$ in (89), the cut-off wavenumber is obtained as

90 $$\begin{aligned} k_c = \frac{-\omega _3\pm \sqrt{\omega _3^2-4\omega _2\omega _4}}{2\omega _4}. \end{aligned}$$

In the case of $q=0$, the cut-off wavenumber is uniquely determined as $k_c=\sqrt{(le+2)/(3le-2)}$. The set up of cellular instability is given by $le=-2$ or 2/3. The positive value of growth rate for small wavenumbers, or long wavelengths, is observed for $le<-2$ and that for large wavenumber, or short wavelength, is observed for $le>2/3$. For $-2<le<2/3$, the growth rate is negative for arbitrary wavenumber.

Although (90) implies the existence of instability at some finite value of wavenumber for $le>2/3$, it should be omitted because the wavenumber is now assumed to be small. In fact, even if the value of *le* is sufficiently large, $k_c$ is not so small: $k_c\rightarrow 1/\sqrt{3}+0$ as $le\rightarrow +\infty$.

The new tendency of cellular instability due to the existence of heat release is characterized by $\omega _3$ in (89). This term promotes the cellular instability accompanied with two cut-off wavenumbers for some range of *le*. The stability boundary of *le* for the cellular instability is obtained from (90) as follows.

91 $$\begin{aligned} le_2<le<le_3 \quad\qquad\qquad {\text {(no cut-off wavenumber)}}, \end{aligned}$$

92 $$\begin{aligned} le< le_1,&\quad le> le_4 \qquad\qquad {\text {(one cut-off wavenumber)}}, \end{aligned}$$

93 $$\begin{aligned} le_1<le<le_2,&\quad le_3<le<le_4&\quad &{\text {(two cut-off wavenumbers)}}, \end{aligned}$$

with

94 $$\begin{aligned} le_1&= -2-\frac{q\beta }{2}\frac{5+3Pr}{1+Pr}, \end{aligned}$$

95 $$\begin{aligned} le_2 =&  -\frac{2}{3}-\frac{q\beta }{12}\frac{2Pr^3-14Pr^2+23Pr+19}{1+Pr}\\ & -\sqrt{\left( \frac{4}{3}-\frac{q\beta }{12}\frac{2Pr^3-14Pr^2+5Pr-11}{1+Pr}\right) ^2-\frac{\omega _3^2}{12}}, \end{aligned}$$

96 $$\begin{aligned} le_3 =&  -\frac{2}{3}-\frac{q\beta }{12}\frac{2Pr^3-14Pr^2+23Pr+19}{1+Pr}\\ & +\sqrt{\left( \frac{4}{3}-\frac{q\beta }{12}\frac{2Pr^3-14Pr^2+5Pr-11}{1+Pr}\right) ^2-\frac{\omega _3^2}{12}}, \end{aligned}$$

97 $$\begin{aligned} le_4&= \frac{2}{3}-\frac{q\beta }{3}\frac{2+7Pr(1-Pr)+Pr^3}{1+Pr}, \end{aligned}$$

where $le_2$ and $le_3$ are the solutions of $\omega _3^2-4\omega _2\omega _4=0$ and determine the cut-off wavenumber which induces the cellular instability. We note that $le_1=le_2=-2$ and $le_3=le_4=2/3$ for $q=0$.

In the stable range of *le* given by (91), the dispersion relation (89) shows negative values of $\omega$ for arbitrary wavenumber and the perturbations disappear in time. On the other hand, in the case of (92), the positive value of growth rate for small wavenumbers is observed for $le<le_1$ and that for large wavenumbers is observed for $le>le_4$. Besides, the new feature of instability due to the existence of heat release is observed in the range of (93). The increase of *Pr* narrows the range of (93), where two cut-off wavenumbers exist, and decreases the values of $k_c$. The cases of $le> le_4$ and $le_3<le<le_4$ are omitted because of the assumption $k^4\ll 1$. In these cases, the cut-off wavenumbers does not take small values.

**Figure 5:**
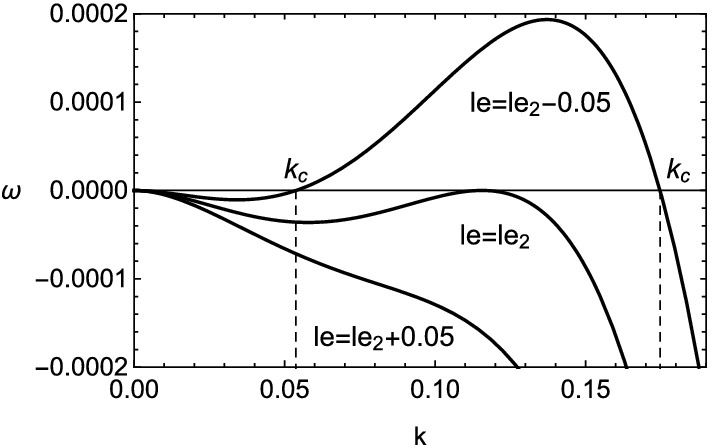
Growth rate $\omega$ v.s. wavenumber *k* for several values of *le*, with $\beta =10$, $q=0.1$ and $Pr=0$. In this case, $le_2\approx -4.327$ and $k_c\approx 0.0538,0.175$.

**Figure 6:**
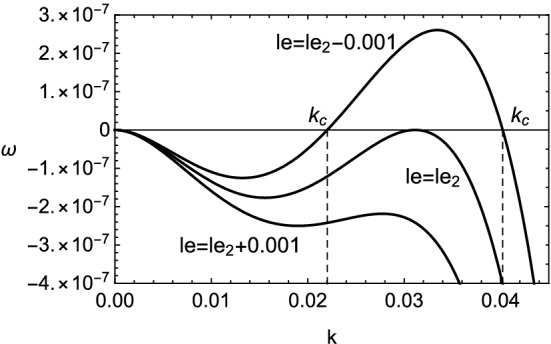
The same as Fig. 5 but for $Pr=3/4$. In this case, $le_2\approx -4.06$ and $k_c\approx 0.022,0.0402$.

**Figure 7:**
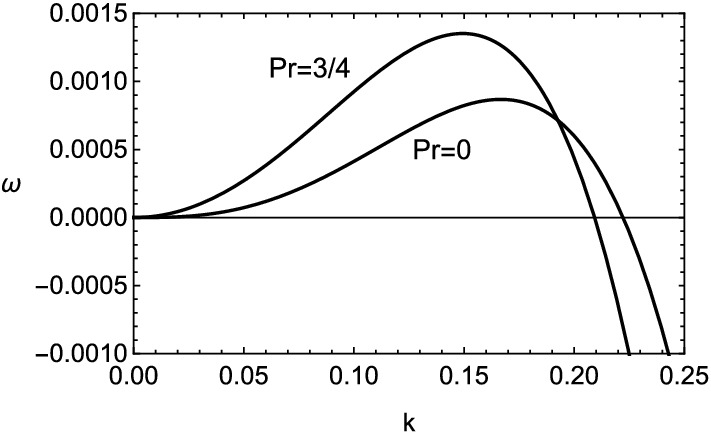
Growth rate $\omega$ v.s. wavenumber *k* for two values of Prandtl number $Pr=0$ and 3/4, with $\beta =10$, $q=0.1$ and $le=le_1(=-4.5)$.

**Figure 8:**
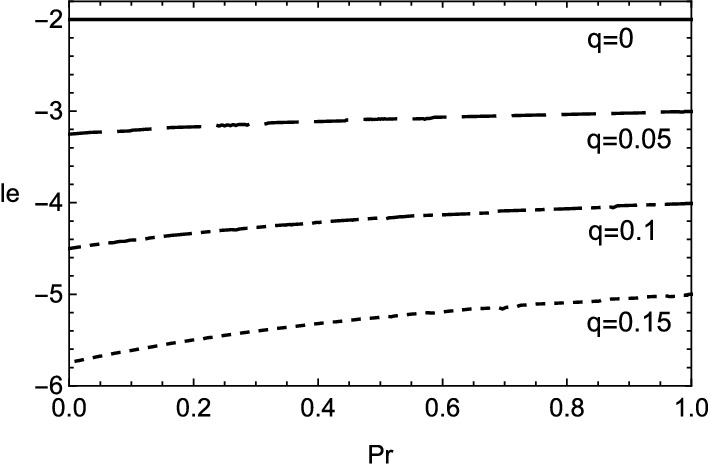
Stability boundary with respect to Lewis number and Prandtl number for small wavenumber with $\beta =10$. The values of *le* less than each curve implies the existence of cellular instability for each value of *q*.

**Figure 9:**
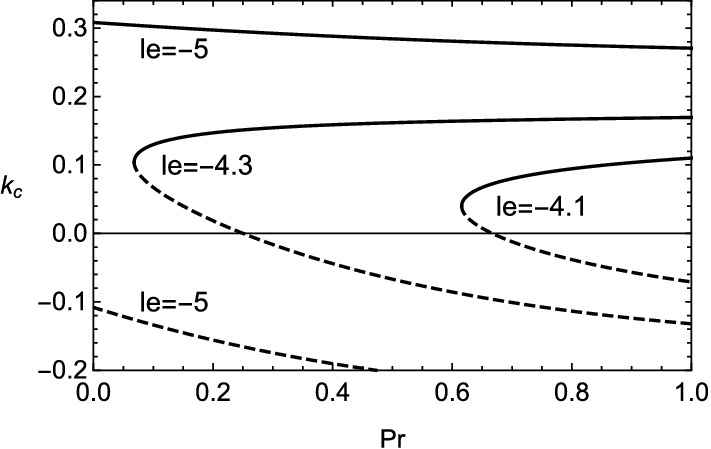
Cut-off wavenumber $k_c$ v.s. Prandtl number *Pr* for various values of reduced Lewis number *le* with $\beta =10$ and $q=0.1$. The solid and dashed lines reflect the signs in (90).

The growth rate in the case of (93) is shown in Figs. 5 and 6. The existence of viscosity raises the value of $le_2$, which implies the Lewis number approaches unity. Therefore, the cellular instability tends to be promoted by the viscosity for small wavenumbers. This fact is consistent with the result obtained numerically in Fig. 2. In the case of (92), it is clear that the viscous effect has two aspects on the instability as plotted in Fig. 7. Although the viscosity enhances the growth rate for small wavenumbers, it shrinks the range of wavenumber where the instability exists. For $k\approx 0$, by setting $\omega =0$ in (89), the stability boundary with respect to the values of Lewis number and Prandtl number is given by $\omega _2=0$ and shown in Fig. 8. The destabilization due to the viscosity for small wavenumbers is easily comprehended from Fig. 8. Finally, the dependence of cut-off wavenumber on the Prandtl number is shown in Fig. 9. It is apparent that, among two cut-off wavenumbers, the smaller one is reduced due to the viscosity. In contrast, as for the larger cut-off wavenumber, the rise of *Pr* brings either increase or decrease of it subject to the values of *le*.

## Numerical study

### Discretization

In this section, the numerical solutions of governing Eqs. (1)–(5) are computed by use of the TVD-MacCormack scheme^38–44^. For simplicity, the two-dimensional flow field in *x*–*z* plane is considered, where the reaction front propagates in the negative *z*-direction. In order to employ the MacCormack scheme^39,40^, (1)–(5) are rewritten into the conservation form:

98 $$\begin{aligned} \frac{\partial \varvec{U}}{\partial t} + \frac{\partial \varvec{E}}{\partial x} + \frac{\partial \varvec{F}}{\partial z}&= \varvec{S}, \end{aligned}$$

where the flux vectors are

99 $$\begin{aligned} \varvec{U}&= \left( \begin{array}{l} \rho \\ \rho U\\ \rho W\\ \rho T\\ \rho Y \end{array} \right) ,&\quad \varvec{E}&= \left( \begin{array}{l} \rho U\\ \rho U^2 + \frac{1}{\gamma {Ma}^2}\rho T - Pr\left( \frac{4}{3}\frac{\partial U}{\partial x}-\frac{2}{3}\frac{\partial W}{\partial z}\right) \\ \rho UW - Pr\left( \frac{\partial W}{\partial x}+\frac{\partial U}{\partial z}\right) \\ \rho TU - \frac{\partial T}{\partial x}\\ \rho YU - \frac{1}{Le}\frac{\partial Y}{\partial x} \end{array} \right) , \end{aligned}$$

100 $$\begin{aligned} \varvec{S}&= \left( \begin{array}{l} 0\\ 0\\ 0\\ qA\rho Y\exp \left( -\frac{(1+q)^2}{q}\frac{\beta }{T}\right) \\ -A\rho Y\exp \left( -\frac{(1+q)^2}{q}\frac{\beta }{T}\right) \end{array} \right) ,&\quad \varvec{F}&= \left( \begin{array}{l} \rho W\\ \rho UW - Pr\left( \frac{\partial W}{\partial x}+\frac{\partial U}{\partial z}\right) \\ \rho W^2 + \frac{1}{\gamma {Ma}^2}\rho T - Pr\left( \frac{4}{3}\frac{\partial W}{\partial z}-\frac{2}{3}\frac{\partial U}{\partial x}\right) \\ \rho TW - \frac{\partial T}{\partial z}\\ \rho YW - \frac{1}{Le}\frac{\partial Y}{\partial z} \end{array} \right) . \end{aligned}$$

Recalling (8) and (9), the coefficient of reaction term is expressed by $A=q^{-2}\beta ^2\Lambda \exp \left( (1+q^{-1})\beta \right)$. In addition, utilizing the result from burning-rate eigenvalue problem under large-activation-energy asymptotics^3,12,13^, we may estimate $\Lambda \approx q^2T_b/(2Le)$. Therefore, the coefficient *A* is approximated by

101 $$\begin{aligned} A = \frac{1+q}{2Le}\beta ^2\exp \left( (1+q^{-1})\beta \right) . \end{aligned}$$

In this study, the time-splitting method^38^ is used to calculate numerical solutions of (98). Then, (98) is divided into two one-dimensional problems corresponding to *x*- and *z*-directions:

102 $$\begin{aligned} \frac{\partial \varvec{U}}{\partial t} + \frac{\partial \varvec{E}}{\partial x} = \frac{\varvec{S}}{2},\quad \frac{\partial \varvec{U}}{\partial t} + \frac{\partial \varvec{F}}{\partial z} = \frac{\varvec{S}}{2}. \end{aligned}$$

The one-dimensional explicit MacCormack scheme is applied to solve each equation in (102), respectively. The MacCormack scheme has second-order accuracy in both time and space. The procedure of discretization is expressed by finite-difference operators $L_x(\Delta t)$ and $L_z(\Delta t)$^46–48^. Each operator consists of two steps: the predictor and corrector steps. For each equation in (102), the predictor step is

103 $$\begin{aligned} & \varvec{U}_{i,j}^p=\varvec{U}_{i,j}^n-\Delta t\left( \frac{\varvec{E}_{i+1,j}^n-\varvec{E}_{i,j}^n}{\Delta _x}-\frac{\varvec{S}_{i,j}}{2}\right) ,\\ &  \varvec{U}_{i,j}^p=\varvec{U}_{i,j}^n-\Delta t\left( \frac{\varvec{F}_{i,j+1}^n-\varvec{F}_{i,j}^n}{\Delta _z}-\frac{\varvec{S}_{i,j}}{2}\right) , \end{aligned}$$

and the corrector step is

104 $$\begin{aligned} & \varvec{U}_{i,j}^{c}=\frac{\varvec{U}_{i,j}^n+\varvec{U}_{i,j}^p}{2}-\frac{\Delta t}{2}\left( \frac{\varvec{E}_{i,j}^p-\varvec{E}_{i-1,j}^p}{\Delta _x}-\frac{\varvec{S}_{i,j}^p}{2}\right) ,\\ &  \varvec{U}_{i,j}^{c}=\frac{\varvec{U}_{i,j}^n+\varvec{U}_{i,j}^p}{2}-\frac{\Delta t}{2}\left( \frac{\varvec{F}_{i,j}^p-\varvec{F}_{i,j-1}^p}{\Delta _z}-\frac{\varvec{S}_{i,j}^p}{2}\right) , \end{aligned}$$

where $\varvec{E}^p$ and $\varvec{F}^p$ are calculated by use of $\varvec{U}^p$. The superscript *n* implies the time step, and the subscripts *i* and *j* imply the spatial steps in *x*- and *z*-directions, respectively.

Each component of flux vectors is discretized in space as follows.

105 $$\begin{aligned} \varvec{U}_{i,j}&= \left( \begin{array}{l} \rho _{i,j}\\ (\rho U)_{i,j}\\ (\rho W)_{i,j}\\ (\rho T)_{i,j}\\ (\rho Y)_{i,j} \end{array} \right) , \nonumber \\ \varvec{E}_{i,j}&= \left( \begin{array}{l} (\rho U)_{i,j}\\ (\rho U^2)_{i,j} + \frac{1}{\gamma {Ma}^2}P_{i,j} - Pr\left( \frac{4}{3}\frac{U_{i,j}-U_{i-1,j}}{\Delta _x}-\frac{2}{3}\frac{W_{i,j+1}-W_{i,j-1}}{2\Delta _z}\right) \\ (\rho UW)_{i,j} - Pr\left( \frac{W_{i,j}-W_{i-1,j}}{\Delta _x}+\frac{U_{i,j+1}-U_{i,j-1}}{2\Delta _z}\right) \\ (\rho TU)_{i,j} - \frac{T_{i,j}-T_{i-1,j}}{\Delta _x}\\ (\rho YU)_{i,j} - \frac{1}{Le}\frac{Y_{i,j}-Y_{i-1,j}}{\Delta _x} \end{array} \right) , \end{aligned}$$

106 $$\begin{aligned} \varvec{S}_{i,j}&= \left( \begin{array}{l} 0\\ 0\\ 0\\ qA(\rho Y)_{i,j}\exp \left( -\frac{(1+q)^2}{q}\frac{\beta }{T_{i,j}}\right) \\ -A(\rho Y)_{i,j}\exp \left( -\frac{(1+q)^2}{q}\frac{\beta }{T_{i,j}}\right) \end{array} \right) ,\nonumber \\ \varvec{F}_{i,j}&= \left( \begin{array}{l} (\rho W)_{i,j}\\ (\rho UW)_{i,j} - Pr\left( \frac{W_{i+1,j}-W_{i-1,j}}{2\Delta _x}+\frac{U_{i,j}-U_{i,j-1}}{\Delta _z}\right) \\ (\rho W^2)_{i,j} + \frac{1}{\gamma {Ma}^2}P_{i,j} - Pr\left( \frac{4}{3}\frac{W_{i,j}-W_{i,j-1}}{\Delta _z}-\frac{2}{3}\frac{U_{i+1,j}-U_{i-1,j}}{2\Delta _x}\right) \\ (\rho TW)_{i,j} - \frac{T_{i,j}-T_{i,j-1}}{\Delta _z}\\ (\rho YW)_{i,j} - \frac{1}{Le}\frac{Y_{i,j}-Y_{i,j-1}}{\Delta _z} \end{array} \right) . \end{aligned}$$

For a flux vector $\varvec{E}$, the backward and central differences are applied to the partial derivatives with respect to *x* and *z*, respectively. On the other hand, for $\varvec{F}$, the central and backward differences are applied to the partial derivatives with respect to *x* and *z*, respectively.

To avoid the oscillation of numerical solutions, we introduce a total variation diminishing (TVD) numerical scheme in the corrector step^41–44^. We employ the Causon’s model^44^. Instead of calculating the inner product of flux vectors as done in the original method, we simply extend the TVD scheme for a scalar quantity to the case of vector flux in order to reduce the computation time. By denoting each component of a flux vector $\varvec{U}$ as $\varvec{U}=(U_k)\;(k=1,2,3,4,5)$, each component of TVD vectors $\varvec{TVDx}=(TVDx_k)$ and $\varvec{TVDz}=(TVDz_k)$ is expressed by

107 $$\begin{aligned} TVDx_{k,i,j}&= \left( G(r_{x\;k,i,j}^+)+G(r_{x\;k,i+1,j}^{-})\right) \Delta U_{k,i+\frac{1}{2},j}-\left( G(r_{x\;k,i-1,j}^+)+G(r_{x\;k,i,j}^{-})\right) \Delta U_{k,i-\frac{1}{2},j}, \end{aligned}$$

108 $$\begin{aligned} TVDz_{k,i,j}&= \left( G(r_{z\;k,i,j}^+)+G(r_{z\;k,i,j+1}^{-})\right) \Delta U_{k,i,j+\frac{1}{2}}-\left( G(r_{z\;k,i,j-1}^+)+G(r_{z\;k,i,j}^{-})\right) \Delta U_{k,i,j-\frac{1}{2}}, \end{aligned}$$

where

109 $$\begin{aligned} & \Delta U_{k,i+\frac{1}{2},j}  = U_{k,i+1,j} - U_{k,i,j} ,\quad \Delta U_{k,i-\frac{1}{2},j} = U_{k,i,j} - U_{k,i-1,j},\\ &  \quad r_{x\;k,i,j}^+ = \frac{\Delta U_{k,i-\frac{1}{2},j}}{\Delta U_{k,i+\frac{1}{2},j}},\quad r_{x\;k,i,j}^- = \frac{1}{r_{x\;k,i,j}^+}, \end{aligned}$$

110 $$\begin{aligned} & \Delta U_{k,i,j+\frac{1}{2}}= U_{k,i,j+1} - U_{k,i,j} ,\quad \Delta U_{k,i,j-\frac{1}{2}} = U_{k,i,j} - U_{k,i,j-1},\\ & \quad r_{z\;k,i,j}^+ = \frac{\Delta U_{k,i,j-\frac{1}{2}}}{\Delta U_{k,i,j+\frac{1}{2}}},\quad r_{z\;k,i,j}^- = \frac{1}{r_{z\;k,i,j}^+}, \end{aligned}$$

111 $$\begin{aligned} & G\left( r_{x\;k,i,j}^{\pm }\right) = \frac{{\text {CFL}}_x}{2}(1-{\text {CFL}}_x)\left( 1-\phi \left( r_{x\;k,i,j}^{\pm }\right) \right) ,\\ &  G\left( r_{z\;k,i,j}^{\pm }\right) = \frac{{\text {CFL}}_z}{2}(1-{\text {CFL}}_z)\left( 1-\phi \left( r_{z\;k,i,j}^{\pm }\right) \right) , \end{aligned}$$

112 $$\begin{aligned} \phi (r)&= \left\{ \begin{array}{ll} {\text {min}}(2r,1) &{} (r>0)\\ 0 &{} (r\le 0) \end{array} \right. . \end{aligned}$$

The Courant–Friedrichs–Lewy factors ${\text {CFL}}_x$ and ${\text {CFL}}_z$ are defined by use of sound speed and velocity field as follows^49^.

113 $$\begin{aligned} & {\text {CFL}}_x = (\tilde{c}_s + {\max _{x,z}}|\tilde{U}|)\frac{\Delta \tilde{t}}{\Delta \tilde{x}} = \left( \frac{1}{{Ma}} + {\max _{x,z}}|U|\right) \frac{\Delta t}{\Delta x},\\ &  {\text {CFL}}_z = (\tilde{c}_s + {\max _{x,z}}|\tilde{W}|)\frac{\Delta \tilde{t}}{\Delta \tilde{z}} = \left( \frac{1}{{Ma}} + {\max _{x,z}}|W|\right) \frac{\Delta t}{\Delta z}, \end{aligned}$$

where (6) and (7) are used. Accounting for the above formulae, the finite-difference operators of TVD-MacCormack scheme are given by

114 $$\begin{aligned} L_x(\Delta t)\varvec{U}^n:&\left\{ \begin{array}{ll} \varvec{U}_{i,j}^p=\varvec{U}_{i,j}^n-\Delta t\left( \frac{\varvec{E}_{i+1,j}^n-\varvec{E}_{i,j}^n}{\Delta _x}-\frac{\varvec{S}_{i,j}}{2}\right) \\ \varvec{U}_{i,j}^{c}=\frac{\varvec{U}_{i,j}^n+\varvec{U}_{i,j}^p}{2}-\frac{\Delta t}{2}\left( \frac{\varvec{E}_{i,j}^p-\varvec{E}_{i-1,j}^p}{\Delta _x}-\frac{\varvec{S}_{i,j}^p}{2}\right) +\varvec{TVDx}_{i,j}^n \end{array} \right. , \end{aligned}$$

115 $$\begin{aligned} L_z(\Delta t)\varvec{U}^n:&\left\{ \begin{array}{ll} \varvec{U}_{i,j}^p&{}=\varvec{U}_{i,j}^n-\Delta t\left( \frac{\varvec{F}_{i,j+1}^n-\varvec{F}_{i,j}^n}{\Delta _z}-\frac{\varvec{S}_{i,j}}{2}\right) \\ \varvec{U}_{i,j}^{c}&{}=\frac{\varvec{U}_{i,j}^n+\varvec{U}_{i,j}^p}{2}-\frac{\Delta t}{2}\left( \frac{\varvec{F}_{i,j}^p-\varvec{F}_{i,j-1}^p}{\Delta _z}-\frac{\varvec{S}_{i,j}^p}{2}\right) +\varvec{TVDz}_{i,j}^n \end{array} \right. . \end{aligned}$$

Then, the update of flux vector $\varvec{U}$ is made as follows^50^.

116 $$\begin{aligned} \varvec{U}_{i,j}^{n+1} = L_z\left( \frac{\Delta t}{2}\right) L_x\left( \Delta t\right) L_z\left( \frac{\Delta t}{2}\right) \varvec{U}_{i,j}^{n}. \end{aligned}$$

### Settings

The initial conditions are given as follows. The development of perturbed reaction front is examined by initially imposing small perturbations on a planar front. We use the sinusoidal displacement periodic in *x*-direction, which is given by

117 $$\begin{aligned} d = a\sin (kx), \end{aligned}$$

where *a* is the initial amplitude of displacement and $k=2\pi /\lambda$ is a wavenumber with $\lambda$ the wavelength of perturbations. The initial solutions are given by those of a steady planar flow in (35). Therefore, the initial values of a flux vector $\varvec{U}$ consists of the following components.

118 $$\begin{aligned} \rho&= \left\{ \begin{array}{lll} 1/(1+q{\text {e}}^{z-9-d}) &{} (z{\le } 9+d)\\ 1/(1+q) &{} (z > 9+d) \end{array} \right. ,\quad \rho U = 0,\quad \rho W = 1, \end{aligned}$$

119 $$\begin{aligned}&  \rho T = \left\{ \begin{array}{lll} 1+\gamma {Ma}^2\left( \frac{4}{3}Pr-1\right) q{\text {e}}^{z-9-d} &{} (z{\le } 9+d)\\ 1-\gamma {Ma}^2q &{} (z> 9+d) \end{array} \right. ,\\ &  \rho Y = \left\{ \begin{array}{lll} (1-{\text {e}}^{Le(z-9-d)})/(1+q{\text {e}}^{z-9-d}) &{} (z{\le } 9+d)\\ 0 &{} (z > 9+d) \end{array} \right. . \end{aligned}$$

The perturbations are imposed on the planar reaction front initially located at $z=9$.

As for the boundary conditions, the spatially periodic conditions are employed in the *x*-direction. In the *z*-direction, the far-field condition (20) is used at the upstream edge and zero-gradient conditions are made at both upstream and downstream edges.

We consider the case of $k=0.5$, correspondingly $\lambda =4\pi$. The parameters are set as $\gamma =1.4$, $Ma=0.01$, $\beta =10$ and $a=0.1$. The Lewis number *Le* is calculated by use of (83). The computational domain is arranged for one wavelength of a perturbed front, $\lambda$, in *x*-direction. In the propagation direction, we consider $0\le z\le 10$.

We use the Grid Convergence Index (GCI) method for estimation of discretization error^51–53^. A representative grid size *h* is given by $h=(\Delta x \Delta z)^{1/2}$ because we consider the case of uniform grid. We select three different sets of grid so as to $h_1<h_2<h_3$. The grid refinement factor is defined by the ratio of grid size, which should be greater than 1.3. Let $r_{21}=h_2/h_1$ and $r_{32}=h_3/h_2$. Then, the apparent order *p* of the numerical method is calculated by

120 $$\begin{aligned} p = \frac{1}{\ln (r_{21})}\left| \ln (\varepsilon _{32}/\varepsilon _{21})+q(p)\right| , \end{aligned}$$

where

121 $$\begin{aligned} q(p) = \ln \left( \frac{r^p_{21}-s}{r^p_{32}-s}\right) ,\quad s = {\text {sign}}\left( \varepsilon _{32}/\varepsilon _{21}\right) ,\quad \varepsilon _{32}=\phi _3-\phi _2,\quad \varepsilon _{21}=\phi _2-\phi _1. \end{aligned}$$

In this study, the key variable $\phi$ of simulation is set as $\rho Y$, which is useful to identify the location of reaction front because $Y=0$ on the burned side. The percentage of oscillatory convergence is identified by the number of $s<0$ among entire lattice points. The approximate relative error $e_a^{21}$ and the grid-fine convergence index ${\text {GCI}}_{\text {fine}}^{21}$ are given by

122 $$\begin{aligned} e_a^{21} = \left| \frac{\phi _1-\phi _2}{\phi _1}\right| ,\quad {\text {GCI}}_{\text {fine}}^{21}(p) = \frac{1.25e_a^{21}}{r^p_{21}-1}. \end{aligned}$$

The numerical uncertainty at each lattice point is estimated by use of ${\text {GCI}}_{\text {fine}}^{21}(p_{ave})$ and indicated by error bars in plotting fine-grid solution $\phi _1$, where $p_{ave}$ is an average value of *p* and measures the global order of accuracy.

### Numerical results

**Figure 10:**
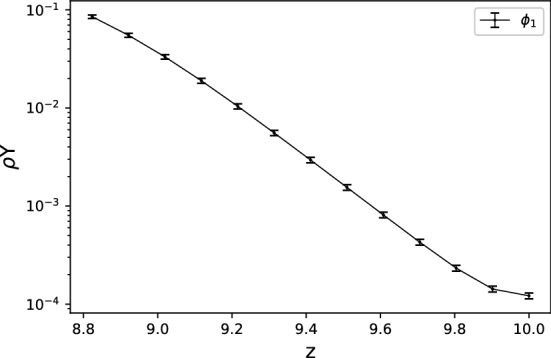
Semi-log plot of fine-grid solution $\phi _1$ with error bars calculated by ${\text {GCI}}_{\text {fine}}^{21}(p_{ave})$ for a $65\times 513$ grid at $y=2\pi$ and *t* = 0.075.

**Figure 11:**
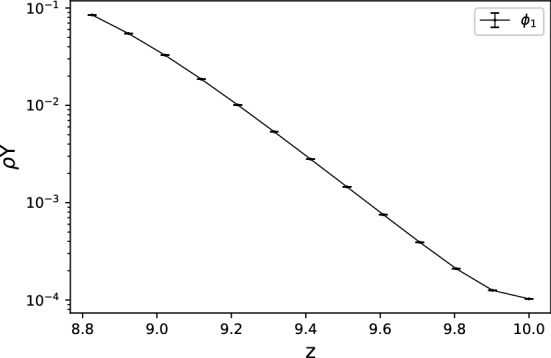
The same as Fig. 10 but for a $65\times 1025$ grid.

**Figure 12:**
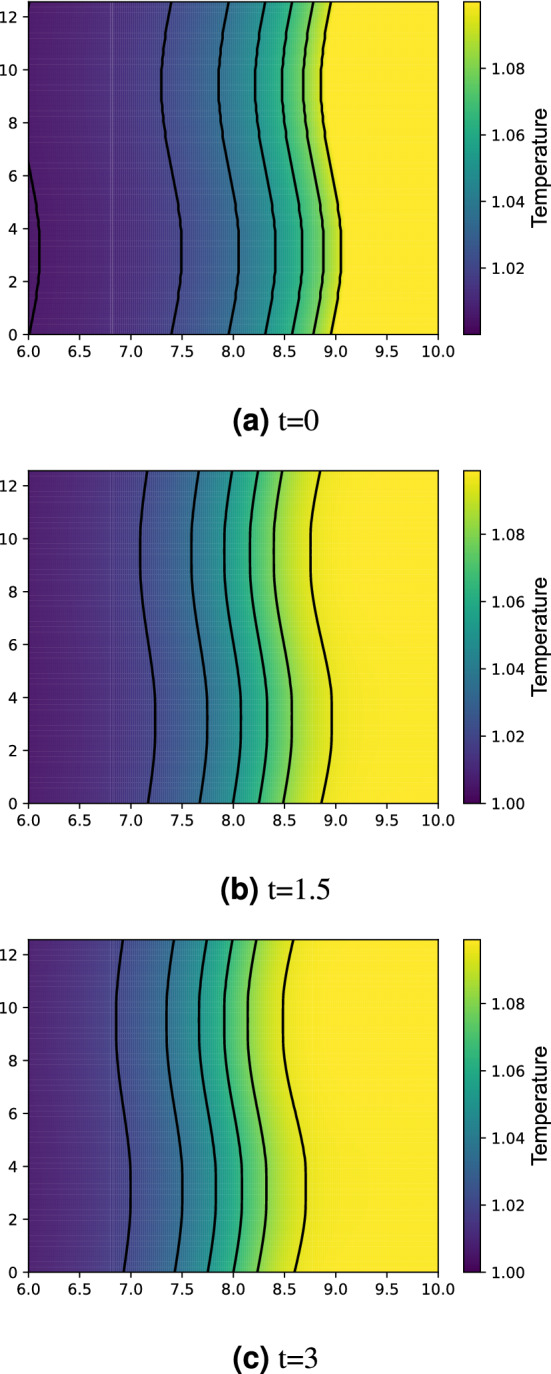
Temperature distribution for $le=-4$ and $Pr=0$.

**Figure 13:**
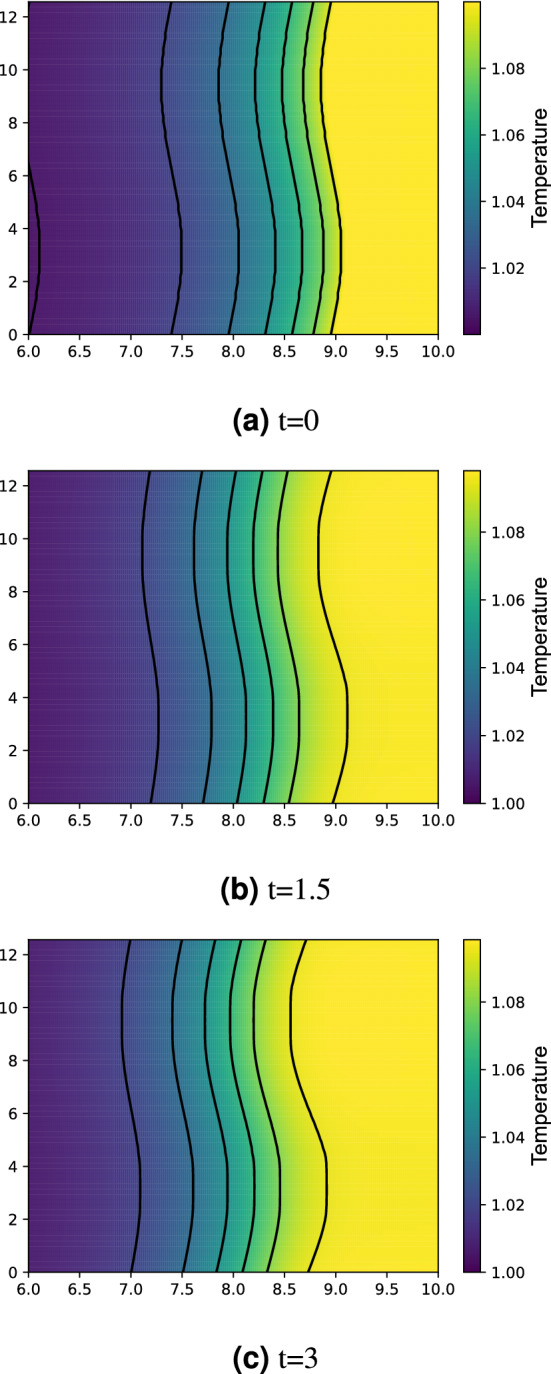
The same as Fig. 12 but for $le=-6$.

**Figure 14:**
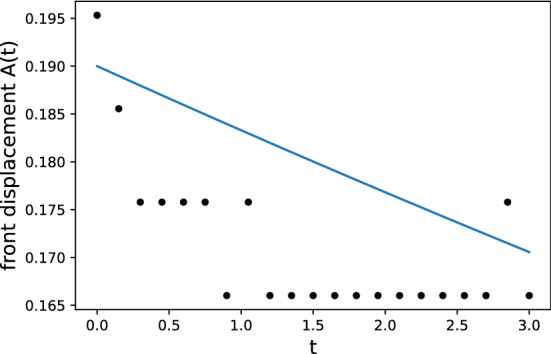
Front displacement for $le=-4$ with a $65\times 1025$ grid and the numerical growth rate is estimated by an exponential curve.

**Figure 15:**
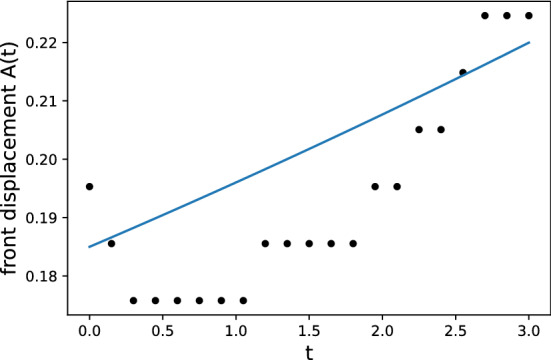
The same as Fig. 14 but for $le=-6$.

The resolution test is shown in Table 1. For $65\times 513$ and $65\times 1025$ grids, the fine-grid solution $\phi _1$ is plotted in Figs. 10 and 11 with error bars calculated by ${\text {GCI}}_{\text {fine}}^{21}(p_{ave})$. In view of GCI, a $65\times 1025$ grid looks like better than others, though the oscillatory convergence occurs at some points and the average apparent order is not near 2, which is a theoretically desirable order of accuracy in the case of MacCormack scheme. By adopting a $65\times 1025$ grid as the spatial resolution, the increments in *x*- and *z*-directions are $\Delta x=4\pi /64\approx 0.2$ and $\Delta z=10/1024\approx 0.01$. The time increment is set as $\Delta t=3\times 10^{-5}$. The temperature distribution is shown in Figs. 12 and 13 for two values of Lewis number, which are chosen so as to show both unstable and stable cases as guessed from Fig. 1. It is possible to estimate the numerical growth rate by considering the front displacement *A*(*t*), which is calculated from the difference of maximum and minimum positions of the front. The front is identified by an isoline $\rho Y = 0.1$. Figures 14 and 15 show the calculated front displacement in time and the estimated exponential curves, $-0.005+0.195\exp (-0.035t)$ and $-0.01+0.195\exp (0.055t)$, which mean the numerical growth rates are $-0.035$ for $le=-4$ and 0.055 for $le=-6$. There is a concern on the decrease of *A*(*t*) at the early stage for $le=-6$. This might indicate that other grids, which would be finer in *z*-direction, are demanded. Besides, the case of small wavenumber has not been examined yet, which should be compared with the analytical result.

**Table 1:** The resolution test is examined for several grids $N_x\times N_z$, where $N_x$ and $N_z$ are the number of lattice points in *x*- and *z*-directions, respectively. All estimation is made at $y=2\pi$ and *t* = 0.075 for every lattice points in *z*-direction.

$N_z$	513		1025	
$N_x$	(17, 33, 65)	(33, 65, 129)	(17, 33, 65)	(33, 65, 129)
$p_{ave}$	1.10185	0.78772	3.61885	7.684307
${\displaystyle \max _z{\text {GCI}}_{\text {fine}}^{21}}(p_{ave})\quad (\%)$	6.8752	8.7668	0.0304172	0.140160
percentage of oscillatory convergence (%)	100	0	1.17302	2.34604

## Discussion

We remark that our results do not contain the Darrieus-Landau instability (DLI), or the hydrodynamic instability. This is because we have employed the boundary conditions across the reaction front, which have been derived systematically under the large activation energy asymptotics, and focused on the pure thermal-diffusive instability. In our case, the density, or the normal velocity, is continuous across the reaction front even for a basic flow, whereas their gradients are discontinuous. Then, the *x*–*y* components of velocity field are also continuous across the front, opposed to their discontinuity in the hydrodynamic model. The discontinuity of density is essential for the existence of DLI. The boundary conditions across a flame front, which reflect the density jump, have been studied in detail based on the multi-scale analysis by introducing the preheat zone inside a flame front^23–26^.

We roughly examine why the DLI drops out in the present study by removing the continuity condition of density, or that of normal velocity, which is expected to lead to the appearance of *O*(*k*) term in the dispersion relation corresponding to the DLI. In order to confirm this, we consider the following solutions of a basic flow, instead of (35), in accordance with those of hydrodynamic model^27,28^.

123 $$\begin{aligned} \bar{\rho }= \left\{ \begin{array}{lll} 1 &{} (\xi <0)\\ 1/(1+q) &{} (\xi > 0) \end{array} \right. ,\quad \bar{W} = 1/\bar{\rho } . \end{aligned}$$

In this case, boundary conditions (62) and (63) are changed into

124 $$\begin{aligned} \frac{\partial W'}{\partial \xi }\Big |_{-}+\frac{\partial \rho '}{\partial \xi }\Big |_{-}&=\frac{1}{1+q}\frac{\partial W'}{\partial \xi }\Big |_{+}+\frac{\partial \rho '}{\partial \xi }\Big |_{+}(1+q), \end{aligned}$$

125 $$\begin{aligned} U'|_{+}-U'|_{-}&=-q\frac{\partial F}{\partial x},\quad V'|_{+}-V'|_{-}=-q\frac{\partial F}{\partial y}. \end{aligned}$$

We employ solutions (47), (49), (54)–(57) and (59), which remain unchanged in order to couple the thermal-diffusive instability with the hydrodynamic one. Substituting these solutions into boundary conditions (64)–(68), (124) and (125), the dispersion relation is calculated, under the assumption of $\omega \sim k^2\ll 1$ and with $O(q^2)$ terms omitted, as

126 $$\begin{aligned}&2(1+q)\omega ^2+\left( \frac{2}{\beta }-(1+q)\left( 1-2Prk-\frac{1-LePr}{1+LePr}\frac{k}{Le}\right) \right) k\omega \nonumber \\&\quad +qk^3+\left( \frac{2}{\beta }-\frac{1-Le}{Le}-q\left( \frac{1}{Le(1+Le)}+\frac{LePr^2}{1+LePr}\right) \right) k^4 =0. \end{aligned}$$

The dispersion relation (126) gives the following growth rate for small wavenumbers,

127 $$\begin{aligned} \omega =\frac{k}{2}-\left( \frac{1}{\beta (1+q)}+\frac{1}{2Le}+\frac{LePr^2}{1+LePr}\right) k^2, \end{aligned}$$

which possesses *O*(*k*) term, which reflects the influence of hydrodynamic instability. Although this result has been obtained in very rude manner, the intrinsic importance of density discontinuity for the existence of DLI may become apparent. Finally, we note that the present study is restricted to the case of continuous density across the reaction front. Therefore, the DLI does not appear.

## Conclusions

We have investigated how the thermal-diffusive instability is affected by the heat release and viscosity. Instead of introducing the effect of thermal thickness of a flame front, or the preheat zone, we directly dealt with the governing equations in the framework of thermal-diffusive model, or ZFK model. This approach has a merit of understanding the viscous effect on the stability boundary of cellular and oscillatory instabilities in a simple manner: the stability boundary is described in the plane with respect to Lewis number and wavenumber. Our main assumption is the small values of heat release with the continuity condition of density across the reaction front, which is valid in the framework of large activation energy asymptotics. Thanks to this assumption, the coupling of hydrodynamic and thermal-diffusive models becomes possible. As a result, the effect of viscosity is incorporated into the dispersion relation for the pure thermal-diffusive instability, though the DLI does not appear.

Due to the existence of heat release and viscosity, the stability boundary of thermal-diffusive instability is largely changed from that obtained in the previous case of no heat release. The heat release has a stabilizing effect on both of cellular and oscillatory instabilities. In other words, for arbitrary wavenumber, the stable range of Lewis number, where the real part of growth rate of perturbations is negative, is widened as the heat release increases. Such a stabilizing effect is also brought by the rise of Prandtl number, which originates from the Navier-Stokes equation, on the oscillatory instability. However, the effect of viscosity has two opposite aspects on the cellular instability. For some moderate values of wavenumber, the stable range of Lewis number for the cellular instability is extended. On the other hand, for small wavenumbers, the stable range is narrowed by the increase of Prandtl number.

The growth rate of cellular instability has been studied in detail by restricting ourselves to small values of growth rate and wavenumber. In this case, we find that the third order term with respect to wavenumber appears in the reduced dispersion relation due to the existence of heat release. This term brings the new band of Lewis number, where two cut-off wavenumbers exist: in other words, there are two wavenumbers at which the growth rate of perturbations changes its sign. The relation between the Lewis number and the cut-off wavenumber is explicitly derived with the viscous effect included. While the value of smaller cut-off wavenumber is reduced as the Prandtl number increases, the behavior of larger one shows both of the increase and decrease depending on values of Lewis number.

The numerical calculation is also made by use of TVD-MacCormack scheme. The numerical growth rate is estimated for different values of Lewis number with small heat release. For moderate value of wavenumber, the numerical result seems to be consistent with the stability boundary obtained in the asymptotic analysis. However, the case of small wavenumbers has not been checked yet. We need to compare this case with the analytical result.

## Data Availability

No datasets were generated or analysed during the current study.
