# Publisher Correction: Effect of small heat release and viscosity on thermal-diffusive instability

**DOI:** 10.1038/s41598-021-00632-9

**Published:** 2021-10-21

**Authors:** Keigo Wada

**Affiliations:** Articulation Center for High School and University, Kanazawa University, Kakuma, Kanazawa, Ishikawa 920-1192 Japan

Correction to: *Scientific Reports* 10.1038/s41598-021-99163-6, published online 12 October 2021

The original version of this Article contained errors in Equations 24–26.

In Equation 24, “$\left[ M \right]$” was omitted from the HTML version.

In Equation 25, “$\left[ {{\varvec{n}} \times \left( {\varvec{V } \times {\varvec{n}}} \right)} \right]$” was omitted from the HTML version.

In Equation 26, “$\left[ T \right]$” was omitted from the PDF and HTML versions.

$$\begin{array}{*{20}c} { = \left[ Y \right] = \left[ {\left( {{\varvec{n}} \cdot \nabla } \right)\left( {T + \frac{q}{Le}Y} \right)} \right] = 0,} \\ \end{array}$$

now reads:

$$\left[ T \right]\begin{array}{*{20}c} { = \left[ Y \right] =\left[ {\left( {{\varvec{n}} \cdot \nabla } \right)\left( {T + \frac{q}{Le}Y}\right)} \right] = 0,} \\ \end{array}$$

The original Article has been corrected.

